# Non-Proteinogenic Amino Acid β-N-Methylamino-L-Alanine (BMAA): Bioactivity and Ecological Significance

**DOI:** 10.3390/toxins14080539

**Published:** 2022-08-07

**Authors:** Olga A. Koksharova, Nina A. Safronova

**Affiliations:** 1 Belozersky Institute of Physico-Chemical Biology, Lomonosov Moscow State University, 119992 Moscow, Russia; 2 Institute of Molecular Genetics of National Research Center “Kurchatov Institute”, Kurchatov Square, 2, 123182 Moscow, Russia

**Keywords:** algae, cyanobacteria, diatoms, glutamate receptors, LC-MS/MS, nitrogen metabolism, oxidative stress, photosynthesis, proteomics, toxic molecules

## Abstract

Research interest in a non-protein amino acid β-N-methylamino-L-alanine (BMAA) arose due to the discovery of a connection between exposure to BMAA and the occurrence of neurodegenerative diseases. Previous reviews on this topic either considered BMAA as a risk factor for neurodegenerative diseases or focused on the problems of detecting BMAA in various environmental samples. Our review is devoted to a wide range of fundamental biological problems related to BMAA, including the molecular mechanisms of biological activity of BMAA and the complex relationships between producers of BMAA and the environment in various natural ecosystems. At the beginning, we briefly recall the most important facts about the producers of BMAA (cyanobacteria, microalgae, and bacteria), the pathways of BMAA biosynthesis, and reliable methods of identification of BMAA. The main distinctive feature of our review is a detailed examination of the molecular mechanisms underlying the toxicity of BMAA to living cells. A brand new aspect, not previously discussed in any reviews, is the effect of BMAA on cyanobacterial cells. These recent studies, conducted using transcriptomics and proteomics, revealed potent regulatory effects of BMAA on the basic metabolism and cell development of these ancient photoautotrophic prokaryotes. Exogenous BMAA strongly influences cell differentiation and primary metabolic processes in cyanobacteria, such as nitrogen fixation, photosynthesis, carbon fixation, and various biosynthetic processes involving 2-oxoglutarate and glutamate. Cyanobacteria were found to be more sensitive to exogenous BMAA under nitrogen-limited growth conditions. We suggest a hypothesis that this toxic diaminoacid can be used by phytoplankton organisms as a possible allelopathic tool for controlling the population of cyanobacterial cells during a period of intense competition for nitrogen and other resources in various ecosystems.

## 1. Introduction

Non-proteinogenic amino acid β-N-methylamino-L-alanine (BMAA) (syn: α-amino-β-methylaminopropionic acid, MeDAP; and 3-N-methyl-2,3-diaminopropanoic acid) was first isolated from the seeds of *Cycas micronesica* K.D.Hill (Cycadaceae) more than half a century ago [1,2]. Non-proteinogenic amino acids (NPAAs) are not naturally encoded genetically and are not contained in the genetic code of any organism; however, they play diverse roles in prokaryotic and eukaryotic organisms. NPAAs are considered to be modified protein amino acids. 

NPAAs are obtained from protein amino acids by adding or removing fragments of CH_2_- or CH- (by lengthening or shortening the carbon chains); amination; hydrogenation and dehydrogenation; and hydroxylation. NPAAs are involved in the biosynthesis of proteinogenic amino acids and serve as a spare form of sulfur and nitrogen. They are a transport form of nitrogen and can perform various protective functions (for example, bind ammonia, which accumulates during the breakdown of proteins) [3,4,5,6]. 

Hundreds of NPAA molecules have been identified as secondary metabolites in various organisms (for instance, more than two hundred of them have been found only in plants) [3]. NPAAs play an important role in the adaptation of organisms to the environment. For example, some NPAAs are phytosiderophores that can chelate Fe^3+^ or Zn^2+^ and increase their uptake by microorganisms [4]. In addition, non-coded amino acids can serve as an organic nitrogen reserve pool in ecosystems [5]. Some non-proteinogenic amino acids (e.g., homoserine and ornithine) are used as intermediates in primary metabolic pathways. Most NPAAs in microorganisms serve as building blocks for the synthesis of small bioactive peptides [6].

The research interest in BMAA arose due to the discovery of a link between chronic exposure to this diaminoacid and the occurrence of neurodegenerative diseases [7,8,9,10,11,12]. The most well-known producers of BMAA are cyanobacteria and eukaryotic microalgae, such as diatoms and dinoflagellates [13]. These photoautotrophic organisms are widely distributed and are known as the primary producers of organic compounds in various aquatic and terrestrial ecosystems. Cyanobacteria and microalgae, being the main source of BMAA in various food chains, pose (due to their periodic blooms) a real threat to consumers of the highest order, including humans [12,13]. 

In addition, BMAA has also been found as a molecular component in antimicrobial peptides produced by *Paenibacillus* spp., widespread facultative anaerobic, edospore-forming bacteria [14], which can also serve as a source of BMAA distribution. Hence, due to the toxicity of BMAA and the wide distribution of BMAA producers in nature, there is a high demand for studies that could uncover the mechanisms underlying this diaminoacid biological action and reveal BMAA’s ecological impact on various food chains and ecosystems, including BMAA-producing communities. 

The principle of selecting papers for this review was based on the following: (1) papers by groups with many years of research experience in the field of BMAA; (2) papers devoted to the most problematic (least studied) issues, for example: (a) ways of biosynthesis of BMAA: different hypotheses; (b) glutamate receptors (GluRs) of plants and cyanobacteria and their role in the binding of BMAA; and (c) the effect of BMAA on nitrogen metabolism in eukaryotic and prokaryotic cells, and others; and (3) papers in which genomic, transcriptomic and proteomic research methods of studying the biological action of BMAA were used in connection with the topic of the Special Issue “Multi-Omics Study of Marine Toxins”. 

This review analyzes and interprets experimental data on the biological activity of BMAA and presents the future prospects for BMAA environmental research. A new aspect, not previously discussed in any reviews, is the effect of this amino acid on cyanobacterial cells. The purpose of this review is to introduce interested researchers to the main current directions of BMAA studies.

## 2. BMAA Producers and Pathways of Biosynthesis

### 2.1. History of BMAA Discovery

The prehistory that led to the discovery of BMAA took place more than fifty years ago on Guam, an island in the western Pacific Ocean [2]. In the early 1950s, an extremely high level of neurodegenerative diseases was registered among the population of Guam; this indicator was 50–100 times higher than the global average [15]. To determine the cause of this anomaly, the researchers examined the brain tissue of Guam residents who died during this time period from the neurodegenerative disease Amyotrophic Lateral Sclerosis/Parkinsonism Dementia Complex (ALS-PDC) and found an interesting fact: brain tissue proteins were bound with a molecule that was identified as BMAA [7,8]. The search for the source of BMAA had been started. 

However, only ten years later, in 1967, BMAA was finally (for the first time) isolated from the seeds of queen sago (*Cycas circinalis*) [5,6], a plant whose seeds had been used for decades in the local diet of the inhabitants of Guam. Later, in the early 2000s, BMAA was discovered in colonies of symbiotic cyanobacterial species—*Nostoc* and *Anabaena,* which were localized within the coralloid roots of *C. circinalis,* and therefore it was suggested that these symbiotic cyanobacteria are the original producers of BMAA and an indirect cause of profound human neurodegenerative disease (which is now known to be caused by the long-term bioaccumulation of BMAA in brain tissue) [9,10,11,12,16].

### 2.2. BMAA Producers

BMAA can be produced by cyanobacteria and microalgae, such as diatoms and dinoflagellates [13].

#### 2.2.1. Cyanobacteria

BMAA can be synthesized by both free-living and symbiotic species of cyanobacteria [17]. Moreover, BMAA has been found not only in filamentous cyanobacterial species but also in various unicellular cyanobacteria. Cyanobacteria producing BMAA are widespread. They can be found in various types of habitats—marine, freshwater, and terrestrial [18,19,20,21,22,23,24,25,26,27]. Strains of cyanobacteria producing BMAA have been collected all over the world—in South Africa [18], Hawaii [19], India, and Australia [17], Peru [20], Great Britain [21], the United States [22], the Swedish waters of Baltic Sea [23], Portugal [24], and Germany [25].

Unexpectedly, it turned out that isomeric forms of BMAA can also be found in cyanobacteria, such as 2,4-diaminobutyric acid (2,4-DAB) and N-(2-aminoethyl) glycine (AEG). These isomeric amino acids were found in various species of freshwater cyanobacteria: *Anabaena*, *Leptolyngbya* sp., *Oscillatoria* sp., *Merismopedia* sp., and *Microcystis aeruginosa* [28]. In addition, BMAA and its isomers have been identified even in 100-year-old Antarctic cyanobacterial mats that are still preserved in the herbarium of the Natural History Museum in London, UK [29].

#### 2.2.2. Diatoms

While many studies have shown that BMAA is produced by cyanobacteria, only a few studies have addressed this issue in relation to eukaryotic groups of algae. Several studies have shown that BMAA-production occurs in marine and fresh-water diatoms [30,31,32,33]. 

The first proof of the fact that BMAA is not only produced by prokaryotic cyanobacteria but can also be synthesized by eukaryotic organisms, such as diatoms, was provided in 2014 by L. Jiang and colleagues in the laboratory of Ulla Rasmussen [30]. In the mentioned study, BMAA was detected in nanogram amounts (1.0–3.8 ng/g dry weight) in six different axenic diatom cultures, viz. *Navicula pelliculosa* (CCAP 1050/9), *Thalassiosira* sp. (CCAP 1085/15), *Achnanthes* sp. (CCAP 1095/1), *Skeletonema marinoi* SAAE08603, *Skeletonema marinoi* ST28, and *Proboscia inermis* (CCAP 1064/1). In addition, BMAA was also found in field samples of plankton that were collected on the west coast of the Swedish Baltic Sea and in which marine diatoms were found in overly representative amounts. 

In the following years, several more studies showed that both marine and freshwater diatoms can produce BMAA [31,32,33]. Four marine diatoms, *Phaeodactylum tricornutum*, *Chaetoceros* sp., *Chaetoceros calcitrans* and *Thalassiosira pseudonana* were studied [31], and for all four species of diatoms, a direct dependence of the concentration of the total amount of soluble BMAA and DAB on the growth phase rate was demonstrated. The highest concentration of BMAA was found during the stationary growth phase of all these studied marine species. This finding allowed the authors to assume that BMAA is a secondary metabolite of diatoms. Later, in another work [32], it was revealed that freshwater diatoms can also produce BMAA, as well as its isomers AEG and 2,4-DAB.

#### 2.2.3. Dinoflagellates

Dinoflagellates are known as a widespread group of plankton living in both marine and fresh waters. Thus far, only one paper [34] has reported that a laboratory-grown culture of dinoflagellate *Gymnodinium catenatum* is capable of producing BMAA. It should be noted that BMAA was found in dinoflagellates in a considerably high level (0.457 ± 0.186 µg/g DW). More research is needed to confirm this discovery.

#### 2.2.4. Bacteria

In 1975, during the search for new antibiotics, BMAA was discovered as a molecular component of peptides that were found to be produced by *Paenibacillus pulvifaciens*, a widespread facultative anaerobic endospore-forming bacterium [14]. Then, in 2014, peptides of a similar structure (viz. with a BMAA in the structure) were isolated from *Paenibacillus larvae,* another species within the *Paenibacillus* genius [14].

*Paenibacillus* spp. is a widespread group of bacteria (more than 200 species of *Paenibacillus* spp. are known), which is the cause of a highly infectious disease of bees, called American foulbrood. The fact that BMAA was found in the structure of peptides synthesized by *Paenibacillus* spp. should be taken into account, considering the common distribution of these bacteria and the fact that some species of *Paenibacillus* spp. have been detected even in food groceries and human interstitial fluids [14]; therefore, the presence of peptide-associated BMAA may pose a hidden threat to consumer (in case BMAA is released) and should be thoroughly studied in the future.

#### 2.2.5. Plants

Can higher plants synthesize BMAA? We cannot answer this question with certainty as our knowledge on this issue is still limited. In only one study, BMAA was found in various tissues of plant *Cycas micronesica* (a plant that is known to have the ability to form a symbiosis with cyanobacteria); however, at the time of the study (according to the authors) did not have symbiotic strains of cyanobacteria on the roots [35]. It was stated that *C. micronesica* seedlings were grown without endophytic cyanobacterial symbiosis. During the growth period, BMAA was quantified at different time points in various parts of the plant. The amount of BMAA increased by 79% during the nine months of seedling growth, and the root tissue contained 75% of the total amount of BMAA [35].

However, a number of questions arise in connection with these findings. How is BMAA synthesis related to plant age and to (laboratory or natural) growth conditions? Are only *Cycas micronesica* able to synthesize this amino acid? Or can other higher plants also do this? Without a doubt, it is necessary to conduct additional research on this issue in order to find an answer to the question: can plants really synthesize and accumulate BMAA in the absence of cyanobacteria?

Summing up all the above, we can state that the current experimental data have shown that various photoautotrophic organisms can produce BMAA, as well as some strains of bacteria. The ability of various species of cyanobacteria and microalgae to synthesize BMAA in a wide range of concentrations—from nanograms to thousands of micrograms per gram of dry weight [17,30,31]—may indicate the biological significance of the role that this molecule plays in the life of these species [36,37,38]. What kind of role could it be? More research is needed to find the answer and confirm it.

### 2.3. Bioaccumulation and Biomagnification

In the last decade, researchers have demonstrated that BMAA, which is synthesized by phytoplankton in aquatic ecosystems, is further transmitted along the food chain to zooplankton and other invertebrates (mussels, oysters, and shrimps) and eventually accumulates in the brain and muscle tissue of fish, as well as in the brains of dolphins (see Section 4.2) and chicken tissues [14,23,39,40,41,42,43,44,45] (Table 1).

**Figure 1:**
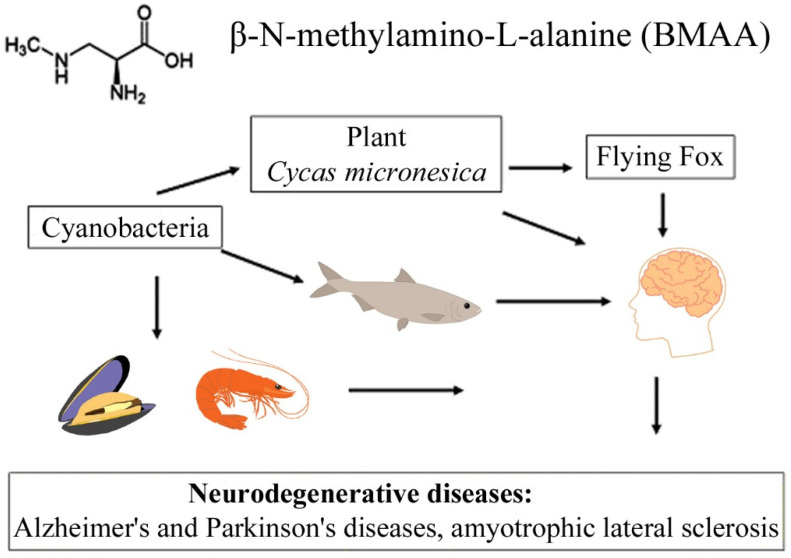
This diagram shows the bioaccumulation pathways of BMAA.

The transfer of BMAA from an aquatic ecosystem to a terrestrial ecosystem can occur, as was demonstrated in [43,44,45], and is caused by irrigation of fields by spraying, in which BMAA accumulates in plant seeds and plant tissues. Finally, passing through various food webs, this amino acid eventually reaches higher animals, including humans [10,11,22,23,40,45] (Figure 1). Protein-associated BMAA molecules were found in significant concentrations (31–356 µg/g, [16]) in the brain tissues of patients who died from ALS-PDC and Alzheimer’s disease (AD) [11,16]. 

A convincing example is the bioaccumulation of BMAA in the food chains of the Guam ecosystem [10,12]. It was found that, in this food chain, concentrations of free and protein-bound BMAA increased drastically during the transition from cyanobacteria (0.3 µg/g and 72 µg/g) to cycad seeds (9 µg/g and 89 µg/g), and then to the Micronesian flying fox *Pteropus mariannus* (3556 µg/g and 146 µg/g) [10]; and an even higher concentration of protein-bound BMAA (627 µg/g) was detected in the postmortem brain tissue of ALS patients as a result of biomagnification [10]. 

It is clear that the facts regarding the bioaccumulation of BMAA are alarming and further research should be conducted to study the mechanisms of synthesis of this molecule, to detect the conditions in which this synthesis occurs, and to clarify the biological role of this compound in the life of organisms producing BMAA.

### 2.4. Biosynthesis Pathways of BMAA: Different Hypothesis

There are several hypotheses about the possible ways of BMAA biosynthesis [14,38,46,47]. BMAA can occur in bacterial cells as a result of cellular metabolic processes [14,38]. Nunn and Codd discussed possible metabolic pathways involved in the biosynthesis of BMAA in cyanobacteria [38]. The authors suggested that BMAA can be formed by methylation of 2,3-diaminopropanoic acid (2,3-DAP) within a macromolecular structure of the multi-enzyme complexes and subsequently released in free form. The first hypothesis was put forward in 2003 by Brenner et al., who suggested a possible pathway for the synthesis of BMAA in the *Cycas rumphii* plant, which was supported by the analysis of expressed sequence tags (EST) from *C. rumphii* [46]. 

The authors suggested that, due to the structural similarity of BMAA with other beta-substituted alanines, such as phosphoserine, cysteine, o-acetylserine, or β-Cyano-L-alanine, alanine can be used for the biosynthesis of BMAA in a two-stage reaction. The first stage of the reaction is the transfer of NH_3_ to the β-carbon site of alanine that is preformed by a cysteine synthase-like protein. The authors identified two candidate genes, which encode cysteine synthase (Gene Bank accession numbers CB089577 and CB092214). The second stage of the reaction consists of the methylation of the beta-amino group of beta-substituted alanine. 

The EST library contains two genes encoding potential methyltransferases: caffeic acid O-methyltransferase II and caffeoyl-CoA 3-O-methyltransferase, which are encoded by CB091906 and CB090738, respectively. These genes are necessary in order to catalyze the second stage of BMAA biosynthesis [46]. Later, in the first review [13], which was devoted to discussing the biological roles of BMAA, it was suggested that the search for homologous genes in the genomes of cyanobacteria and diatoms is of interest, since it can contribute to elucidating the mechanisms of BMAA synthesis in these organisms. 

Most recently, the first such genomic study was conducted [47]. In this interesting study, the authors used bioinformatics tools to explore hypotheses regarding BMAA biosynthesis in cyanobacteria by assessing the presence or absence of enzymes in six known potential metabolic pathways in 130 cyanobacterial genomes. It has been shown that most of the enzymes involved in the pathways leading to the putative precursor of BMAA (2,3-diaminopropanoic acid, 2,3-DAP) in other species have not been detected in cyanobacteria. Only genes encoding SbnA and SbnB were found in a limited subset of cyanobacterial species. 

Due to the coordinated action of these proteins, the biosynthesis of 2,3-DAP occurs in Gram-positive bacteria *Staphylococcus aureus* Rosenbach 1884. The authors emphasized the potential physiological role of 2,3-DAP in the formation of siderophores in some cyanobacteria species and showed that the *pam* gene cluster responsible for directing the biosynthesis of the peptide-bound BMAA in *Paenibacillus larvae* (*Paenibacillaceae*), was not found in the genomes of 130 species of cyanobacteria and was also not found in 93 genomes of *Paenibacillus* (except of *P. larvae*). 

This study also showed that the presence in some cyanobacteria species of genes presumably encoding the enzymes 2,3-diaminopropionate ammonia lyase (DAPAL, EC 4.3.1.15) and reactive intermediate deaminase A (RIDA, EC 3.5.99.10) may explain the inability to detect 2,3-DAP in analytical studies. DAPAL is a prokaryotic type II PLP-dependent enzyme that catalyses the degradation of R- and S-forms of 2,3-DAP to 2-aminoacrylate and ammonium.

The biosynthesis of 2,3-DAP in cyanobacteria seems to be either limited to a small subset of cyanobacterial species, or there may be many additional ways of biosynthesis of this amino acid. The authors believe that it is also possible that cyanobacteria synthesize BMAA using a pathway or pathways that have not yet been discovered.

**Figure 2:**
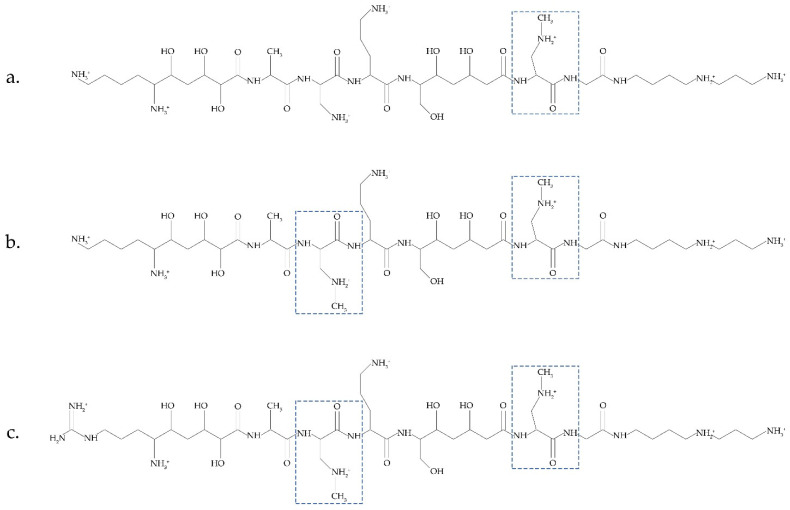
Antimicrobial peptides (galantin (**a**) and paenilamicins (**b**,**c**)), which include BMAA (modified figure from [14]).

Another possible source of BMAA was recently discussed in [14]. Bacteria *Paenibacillus pulvifaciens* and *Paenibacillus larvae* contain antimicrobial peptides (galantins and paenilamicins), which include BMAA (Figure 2). In cells of *P. larvae*, BMAA occurs within an NRPS-multienzyme complex by the methylation of the 2,3-DAP residue. A cluster of *pam* genes necessary for the biosynthesis of paenilamicins was identified [48]. Nunn and Codd suggested that free BMAA does not occur in the cells of *P. larvae*. This bacterium is responsible for a highly infectious disease of bees. 

A metabolic turnover of paenilamicin antibiotics and their hydrolysis by peptidases can occur in the intestines of honeybee larvae. As a result, a free BMAA is released. Considering that *Paenibacillus* species are always present in the environment and are used agriculturally and that some *Paenibacillus* species infect human food and have even been isolated from human body fluids [14], we can conclude that it is necessary to investigate these bacteria and their peptides in detail as another important source of BMAA. Considering that cyanobacteria also synthesize biologically active toxic peptides [49], it will be interesting to clarify whether cyanobacteria can also have BMAA as a peptide component.

It is important to keep in mind the possibility of spore formation by many bacteria, including *Paenibacillus* spp. There is a medical hypothesis (the so-called “spore hypothesis”) that explains both early-onset and late-onset Parkinson’s disease [50]. There is a hypothetical probability of the formation of BMAA-endospores from *Paenibacillus* spp. that can penetrate into the central nervous system [14]. In addition, we should consider the possibility of the existence of other BMAA-producing bacteria that are part of the human microbiome [14]. 

The production of BMAA by representatives of the intestinal microbiota has been hypothesized as another possible pathway of chronic exposure to BMAA [51,52]. This assumption should be investigated. One of the potential candidates for the role of such an “internal enemy” is *Melainabacteria,* a non-photosynthetic phylogenetic clade of divergent cyanobacteria identified as representatives of the human gut microbiota [53,54]. These hypotheses under discussion are waiting for their research and confirmation or refutation.

## 3. BMAA Identification in Cells and Natural Environment Samples

### 3.1. Experimental Approaches and Methods Used for BMAA Identification

To study the biological role of BMAA in the life of its producers, the effects of this amino acid on living cells, and the levels of accumulation of this toxin in food chains, in water, and in other natural samples, it is necessary to have sufficiently sensitive research methods, which permit obtaining reproducible results. Analytical procedures (selectivity and sensitivity of the method as well as quality control) play a crucial role in the identification of BMAA in biological samples (Table 1). They help to assess the relationship between BMAA and neurodegenerative diseases and determine the ways of human exposure [18,21,23,55,56,57,58,59,60,61,62]. Initially, BMAA was identified by ion-exchange chromatography [1]. Later, other methods were used to identify BMAA. These are high-performance liquid chromatography (LC) with fluorescence detection [17,21,63], gas chromatography/mass spectrometry (GC/MS) [64,65], capillary electrophoresis [66], ultra-high liquid chromatography with tandem mass spectrometry (UHPLC/MS/MS) [23,26] and differential mobility MS/MS [67,68]. 

Nuclear magnetic resonance (^1^H NMR) has also been used to detect BMAA in natural water samples [69]. In this work, the amino acid (without derivatization) was extracted from the samples by solid-phase extraction. The detection limit for BMAA was five μg·mL^−1^, which is three orders of magnitude less sensitive than the LC/MS/MS determination. The LC/MS/MS method with derivatization is currently the most reliable for detecting BMAA in biological samples [70]. 

Many procedures include the preliminary preparation of BMAA derivatives using 6-aminoquinolyl-N-hydroxy succinimidyl carbamate, propyl chloroformate (PCF), 9-fluorenylmethyl chloroformate, and dansyl chloride [10,11,18,23,71,72,73,74]. The hydrophobicity of derivatives increases the retention time and, consequently, increases the efficiency of separation of products on reverse-phase carriers (RP-HPLC method). The high molecular weight of the derivatives improves the signal-to-noise ratio and the ionization reaction of electrospray [66]. A higher sensitivity for detecting BMAA derivatives based on fluorescence has been demonstrated [17,21]. 

Methods for determining BMAA and its isomers are being improved and developed. A new analytical approach was recently developed to detect BMAA and its isomers at ultra-trace levels in surface water samples [61]. The authors applied pre-column aqueous derivatization with 9-fluorenylmethyl chloroformate (FMOC-Cl) to the targeted amino acids in the surface water matrix. This is a fast and sensitive method for monitoring BMAA, AEG, β-amino-N-methyl-alanine (BAMA), and DAB in surface water. In [75], a simple LC-MS/MS method was developed to determine the presence of both D- and L-isomers of BMAA in complex samples. The sample preparation procedure included enzymatic hydrolysis followed by derivatization with (+)-1-(9-fluorenyl)-ethyl chloroformate (FLEC). The technique [75] allows determining the presence of BMAA enantiomers and separating them from its structural isomers. Moreover, the derivatization of BMAA enantiomers with FLEC led to the production of diastereomers that can be separated by HPLC with a reversed phase. This experimental method can be applied to the analysis of biological samples to assess the distribution of BMAA isomers in various sources.

The procedure for extracting BMAA from biomass is crucial for detecting BMAA in biological samples. BMAA can be found as free amino acid and in a protein precipitated fraction [9]. There are different methods of protein precipitation [17,19,30,34,70,76] and cation-exchange solid-phase extraction [23,26,77]. The most effective methods of extracting BMAA from cyanobacteria and diatoms are solid-phase extraction [23,26], protein precipitation with acetone [30], and trichloroacetic acid (TCA). The latter method is often used in BMAA studies [10,11] and can generally be considered the best [77].

### 3.2. Methodological Difficulties in BMAA Identification

Special attention should be paid to the interesting and useful critical reviews by Faassen [57], Bishop and Murch [56], and Dunlop et al. [60], in which the authors carefully considered and discussed the methodological problems that arise when determining the presence and amount of BMAA in the studied samples. BMAA can only be reliably detected by using appropriate methods. Elisabeth Faassen, for the first time in 2014, raised the question of the correctness and accuracy of the determination of BMAA in different laboratories. It has been noted that the reported concentrations of BMAA in aquatic systems vary greatly from study to study. Several studies have identified BMAA in all tested samples of cyanobacteria, while others have not found it in any sample [57]. In addition, the concentrations of BMAA in cyanobacteria vary by orders of magnitude in different studies. Similarly, several studies have identified BMAA at higher trophic levels, such as shellfish and fish; however, others have not. Bioaccumulation of BMAA in higher aquatic organisms has been reported. However, for example, BMAA concentrations in the two studies of food-webs conducted thus far are very different: the concentrations recorded for the Baltic Sea (mainly ng/g dry weight (DW)) [23] were several orders of magnitude lower than in Florida (high μg/g to mg/g DW) [22]. Dr. Faassen critically considered the possibility of comparing the results obtained in different research groups. It was important to understand the reasons for the possible errors in the identification of BMAA: errors and inaccuracies arise either because inadequate analytical methods were used or because poor presentation of the analysis results made it impossible to verify the results. Poor analysis, reporting, and numerous errors have shaken the foundations of BMAA research. Clearly, the first steps to assessing the impact of BMAA on humans are the development and use of selective, inter-laboratory validated methods and the correct presentation of analytical reports [57].

Bishop and Murch [56] identified 148 eligible studies of which a positive result for BMAA in one or more samples analyzed was reported in 84% (125 out of 148) of total studies, 57% of studies of hydrophilic interaction liquid chromatography (HILIC), 92% of studies of reverse-phase liquid chromatography (RPLC), and 71% of other studies. The authors emphasized that the greatest discrepancy between the different methods arose from the analysis of cyanobacteria samples, where BMAA was detected in 95% of RPLC studies but only in 25% of HILIC studies. 

Without sufficient published validation of the performance of each method, it is difficult to determine whether each method is suitable for each sample matrix. The importance of developing methods appropriate to their intended use is confirmed by inconsistent reports of BMAA in environmental samples, despite its prevalence in various ecosystems and food webs.

Dunlop et al. in 2021 indicated the possibility that exposure to BMAA in cyanobacterial blooms or food sources may be a risk factor for neurological diseases, which is currently being studied by groups of interdisciplinary researchers in Australia, Canada, China, France, Norway, South Africa, Spain, Sweden, and other countries around the world. The authors emphasized the need for the optimization and validation of analytical methods and proposed strategies to improve the implementation and reporting of analytical methods as well as the need for additional and well-performed interlaboratory comparisons to quantify BMAA [60].

## 4. Molecular Mechanisms Underlying BMAA Toxicity

### 4.1. The Effect of BMAA on Various Organisms: Pleiotropic Toxic Effect

**Figure 3:**
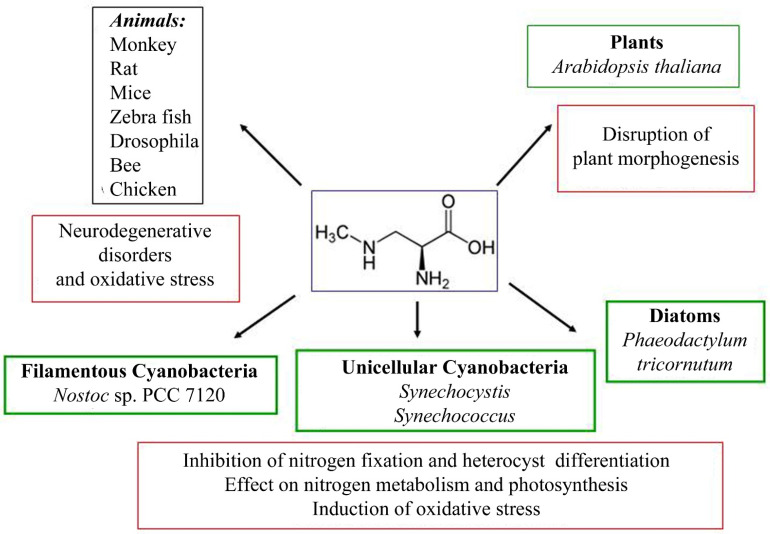
The scheme shows the effects of BMAA on model species of animals and plants, as well as cyanobacteria and diatoms.

The effect of BMAA has been studied mainly on various animal models and human cell cultures due to its harmful consequences for health [78,79,80,81,82,83,84,85,86,87,88,89,90,91,92,93,94,95,96,97]. There are several studies on the effects of BMAA on plant cells [98,99,100,101,102], diatoms [103], and cyanobacteria [27,37,104,105,106,107,108,109,110,111]. The biological actions of BMAA on eukaryotic and prokaryotic cells can be explained by various mechanisms [13,78] (Figure 3). The molecular mechanisms of action of BMAA include binding to glutamate receptors in animals and disruption of the regular activity of motor neurons [82,87,112], participation in protein synthesis and protein binding in animals [113,114,115,116,117,118,119,120,121]. 

BMAA can induce mitochondrial dysfunction in mouse cells [122,123,124]. Recently, the damaging effect of BMAA on rat retina neurons and Müller glial cells has been demonstrated [125]. BMAA binds transition metal ions, such as Zn^2+^, Cu^2+^, and Ni^2+^ [126,127,128,129,130]. BMAA alters the regulation of nitrogen metabolism and photosynthesis in cyanobacteria [36,37,104,105,106,107,108,109]. Exposure to BMAA impairs the morphogenesis process and causes oxidative stress, DNA repair, and apoptosis [107,108,109,131,132,133,134]. The persistent nature of the effects caused by BMAA may be associated with epigenetic changes, since exposure to BMAA reduces global DNA methylation in cells [134]. Below, we discuss the main targets of BMAA action in eukaryotic and prokaryotic organisms.

### 4.2. BMAA Neurotoxicity Hypothesis: BMAA as a Glutamate Receptors Agonist

Many data suggest that BMAA is neurotoxic amino acid. BMAA causes neurological changes in chickens, mice, rats, and dolphins [82,135,136,137] and causes impaired functioning of motor neurons in primates [8,136]. The rate of development of motor dysfunction in primates depends on the amount of BMAA used [8]. BMAA can penetrate the blood–brain barrier [138,139].

The impact of BMAA was assessed by examining transcription signatures using PCR for seven dolphin genes homologous to genes involved in Alzheimer’s disease (AD) and related dementia [137]. The authors demonstrated that gene transcription regulation in dolphin cohorts occurred in parallel with an increase in BMAA concentration. Moreover, dolphins with BMAA exposure equivalent to those reported in AD patients had a 14-fold increase in AD-type neuropathology. The results showed that dolphins develop neuropathology associated with AD, and exposure to BMAA can enhance these processes [137].

A strong toxic effect of BMAA was observed on *Danio rerio* fish. The effect of the toxin on fish embryos causes abnormal formation of the spine axis, rapid muscle contraction in fish larvae, and death [83]. Proteomic analysis revealed seven proteins associated with signaling and recycling of glutamate receptors. All nine other proteins were associated with a violation of protein homeostasis, the formation of reactive oxygen species (ROS), or the death of neuronal cells [140].

BMAA also demonstrates neurotoxic effects on insects. The neurotoxin causes motor disorders and death of the fruit fly (*Drosophila melanogaster*) [141] as well as changes in neurons and high mortality in bees (*Apis mellifera*) [142]. BMAA exhibits neurotoxicity due to competitive binding to various glutamate receptors [143]. The toxin and its carbamate form can act as agonists of various glutamate receptors (N-methyl-D-aspartate (NMDA) receptor, alpha-amino-3-hydroxy-5-methyl-4-isoxazole propionic acid/kainate receptor, and metabotropic glutamate receptor mGluR5). This leads to the increased stimulation and degeneration of motor neurons [112,144]. The addition of BMAA leads to an increase in the intracellular concentration of Ca^2+^ [145,146,147,148]. This, among other factors, leads to the overexcitation of neurons and, ultimately, to cell death.

Glutamate receptors (GluRs) are also found in plants [46,149,150,151,152,153,154,155]. Plants demonstrate coordinated and well-regulated behavior throughout the whole organism, using integrated signaling, communication, and response systems [155]. Phylogenetic analysis using GluRs from metazoan, plants, and bacteria showed that plants are no more closely related to metazoan GluRs than they are related to bacterial GluRs [152]. The number of GluR coding sequences in plants is higher compared to prokaryotes and algae. This allows us to assume the existence of a common land plant GluR ancestor, which went through a series of gene duplications, which led to the development of various specific functions of GluRs [154]. The roles of plant proteins were investigated using various experimental approaches. For example, the genetic approach allowed Brenner and co-authors to find a BMAA-insensitive mutant with a mutation in the gene encoding proteasome-related protein (RPT3), which is involved in glutamate-mediated signaling in plants [46].

Phylogenetic studies have shown that cyanobacterial GluRs are closely grouped with other bacterial homologues, which refutes the possibility that the plant GluRs have a cyanobacterial origin [152]. Phylogenetic analysis also showed that two clusters of cyanobacteria (*Nostocales* versus *Chroococcales* and *Prochlorales*) do not belong to the same group [154,156]. Phylogenetic analysis was performed [156] for the GluR of 17 species of cyanobacteria. This analysis was based on amino acid sequences selected according to the homologous proteins of *Synechocystis* sp. PCC 6803 and *Nostoc* sp. PCC 7120. One cluster includes mainly filamentous cyanobacteria forming heterocysts, which suggests a common origin of GluR in representatives of the genera *Nostoc*, *Anabaena*, *Calothrix*. Another cluster includes unicellular diazotrophic and non-diazotrophic marine and freshwater cyanobacteria [156]. The GluRs of cyanobacteria forming heterocysts make up one cluster, which allows us to hypothesize that this receptor may be involved in the regulation of heterocyst differentiation. Upcoming experimental studies will test this hypothesis.

### 4.3. BMAA and Proteins: Misincorporation and Electrostatic Binding

Another possible mechanism of action of BMAA on the cellular metabolism is its ability to be mistakenly incorporated into proteins [113,114,117,118,121]. In human cells, BMAA is incorporated into proteins instead of L-serine. It was experimentally shown [117] that labeled [^3^H]BMAA was incorporated into proteins of several human cell lines (lung fibroblasts, neuroblastoma cells, and umbilical vein endothelial cells) instead of L-serine, which led to the formation of defective proteins. Improper protein folding, which occurs when BMAA replaces L-serine during protein synthesis, can lead to the formation of prions [118], which can continue the process of neurodegeneration even after BMAA has been eliminated from the body by lymphatic protein recycling. 

BMAA incorporation in proteins leads to improper protein folding and toxicity associated with ALS and AD [115,119,120]. Using a cell-free system, it was shown that BMAA effectively replaces serine and alanine in proteins [114]. Approximately half of the BMAA in the synthesized proteins was released during protein denaturation with sodium dodecyl sulfate and dithiothreitol, and the other half was released only during the acid hydrolysis of proteins. 

Consequently, it has been proven that BMAA is incorporated in the amino acid chain of a polypeptide during its synthesis and is associated with proteins by forming noncovalent bonds [114]. BMAA was detected in the mammalian brain [113]. Polyubiquitinated protein forms were found in the hippocampus region of the brain of rats exposed to BMAA [86], which is part of the limbic system responsible for emotions, long-term memory, and the general orientation of the body in space. Loss of hippocampal volume is an early sign of Alzheimer’s disease.

Recently, Han and co-authors discovered that BMAA is a substrate for human alanyl-tRNA synthetase (AlaRS) and can form BMAA-tRNAAla, avoiding internal proofreading of AlaRS [121]. Moreover, the authors reported that cyanobacterial AlaRS also activates BMAA [121]. It was previously found [157] that exogenous BMAA did not inhibit the growth of heterotrophic bacteria. The authors suggested that BMAA cannot be incorporated into the proteins of heterotrophic bacteria cultured in both rich and poor nutrient media [157]. 

BMAA showed no mutagenic, cytotoxic, or genotoxic effects on several strains of the heterotrophic bacterium *Salmonella typhimurium* [158]. At the same time, the photoautotrophic growth of cyanobacteria was inhibited by exogenous BMAA in micromolar amounts [104,105,106,107,108,109,159]. Moreover, the results of many published studies [57] demonstrate that BMAA was found mainly in protein-associated fractions of cyanobacterial cells. The previously mentioned work by Han et al., 2020 [121] allows us to express the hope that future studies using various bacterial model organisms, including cyanobacteria, can help clarify the question about BMAA incorporation in bacterial proteins.

BMAA can interact with proteins without *de novo* protein synthesis [116]. In this study, the authors have demonstrated that the interaction of BMAA with proteins (b-amylase, catalase, and glutathione S-transferase) reduces the activity of these enzymes. It has been suggested that the interactions of BMAA with proteins are electrostatic in nature. The novel mechanisms of BMAA toxicity may include: (1) BMAA-induced enzyme inhibition; (2) interference with protein folding; and (3) association of BMAA with proteins, especially at sites important for posttranslational modifications. This mechanism requires further study.

### 4.4. BMAA as a Metal-Binding Amino Acid

BMAA can bind transition metal ions (Cu^2+^, Zn^2+^, and Ni^2+^) [126]. It was found that the ability of BMAA to bind Zn^2+^ is significantly higher than that of glutamate. It was revealed that, in the presence of BMAA, the glutamate complex with Zn^2+^ dissociates, and Zn^2+^ binds to a toxic amino acid [126]. 

Zn^2+^ plays an important role in the central nervous system [127], and experimental evidence of the effect of the Zn^2+^ dyshomeostasis and glutamate system disorders in ischemic and neurodegenerative disorders has been provided [129]. Zn^2+^ modulates mainly postsynaptic, ionotropic, and metabotropic receptors [128]. It will be interesting to study the relationship between the properties of BMAA associated with metals and its neurotoxicity.

In the presence of bicarbonate ions, BMAA forms carbamate adducts that exhibit excitatory effects [80,160,161]. Diaz-parga and coauthors (2020) [130] recently discovered using NMR spectroscopy that BMAA interacts differently with divalent metal ions (Mg < Zn < Cu). This property allows BMAA molecules to influence the rate of formation of carbamate products. The authors suggested that, under physiological conditions, the equilibrium between BMAA, bicarbonate ions, and divalent metal ions can change the total population of a specific form of BMAA-ion complex and affect the mechanisms of neurotoxic action of BMAA [130]. There are still few studies of BMAA binding to metals. Given the vital role of metals in the functioning of enzymatic complexes in living cells, this line of research is promising and could be very informative.

### 4.5. The Effect of BMAA on Nitrogen Metabolism in Eukaryotic and Prokaryotic Cells

BMAA affects the synthesis and degradation of glutamine and glutamate in eukaryotic and prokaryotic cells. BMAA inhibits the synthesis or stimulates degradation of glutamine in rat tissues [162] and induces the loss of glutamate by acting on the cystine/glutamate antiport system in mouse cell cultures [163].

Experiments with isotope (L-BMAA-4,4,4-D3,^15^N_2_)-labeled BMAA in cells of the nondiazotrophic cyanobacterium *Synechocystis* PCC 6803 demonstrated that the primary amino group of BMAA can be transferred to other amino acids [37]. In particular, the label from BMAA can be redistributed between free glutamine and glutamic amino acids. Transamination of BMAA in *Synechocystis* PCC 6803 is catalyzed by glutamine oxoglutarate aminotransferase. According to the authors [37], this may indicate the involvement of this enzyme in BMAA metabolism in cyanobacteria cells.

Exogenous BMAA in a small amount (5 µM) does not affect the growth of *Synechococcus* sp. strain TAU-MAC 0499 but affects nitrogen assimilation in this unicellular cyanobacterium [110]. In the diatoms *Phaeodactylum tricornutum* and *Thalassiosira weissflogii*, extracellular ammonia was detected in media of both species at the 0.5 µM of exogenous BMAA [103]. These data suggest that BMAA interferes with nitrogen metabolism also in diatoms, possibly by inhibiting ammonium assimilation via the GS/GOGAT pathway.

Recently, completely new results have been obtained on the regulatory effect of exogenous BMAA on the main metabolic pathways of filamentous nitrogen-fixing cyanobacteria [104,105,106,107,108,109]. We will focus in more detail on these new results as they are important for understanding the molecular mechanisms of the action of BMAA on the cells of cyanobacteria-producers of this diaminoacid and are presented for the first time in this review. 

**Figure 4:**
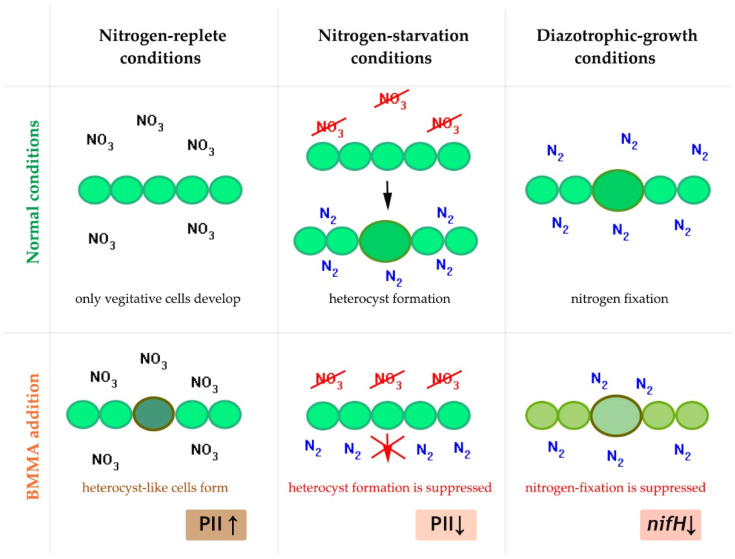
Illustration of the regulatory effect of BMAA on cell differentiation and nitrogenase activity in diazotrophic cyanobacteria under various growth conditions [109]. BMAA up-regulates protein PII in nitrogen-replete conditions [108] and down-regulates protein PII in conditions of nitrogen starvation [107]. In mature heterocysts BMAA strongly inhibits the expression of the nifH gene in diazotrophic-growth conditions [105].

Using transcriptomic [105,106] and proteomic [107,108,109] experimental approaches, the authors showed that BMAA demonstrates a noticeable regulatory effect in diazotrophic cyanobacteria that form heterocysts—special differentiated cells in which the fixation of atmospheric nitrogen occurs (Figure 4). The functional state and development of diazotrophic cyanobacteria depend on the growth conditions. If nitrogen in the form of nitrate or ammonium is present in the growth medium, cyanobacterial filaments contain only vegetative cells (Figure 4). During diazotrophic growth in a nitrogen-free medium, these cyanobacteria use specialized cells, heterocysts that protect the anaerobic nitrogen fixation enzyme nitrogenase from oxygen inactivation (Figure 4). Heterocysts supply neighboring vegetative cells with nitrogenous compounds and, in turn, receive reduced carbon compounds from vegetative cells [164]. 

There is a short transition period (24–48 h) between these two main physiological states (nitrogen replete and diazotrophic growth conditions) when cyanobacteria form heterocysts from vegetative cells (Figure 4). This process of cell differentiation is triggered by intracellular signals of nitrogen deficiency (nitrogen starvation) and is under genetic control and depends on several regulatory proteins [164]. The differentiation of heterocysts involves many important intracellular events in vegetative cells before they become mature heterocysts.

BMAA demonstrated the considerable effect on cyanobacterial cells in all these three physiological conditions (Figure 4) [104,105,106,107,108,109]. The studies were conducted using a model strain of cyanobacteria *Anabaena* (*Nostoc*) sp. PCC 7120, for which there is more genetic information. In the botanical literature, this strain is associated with *Anabaena* spp. [165,166,167]. However, over the past decade, this strain has been referred to as *Nostoc* sp. PCC 7120 in the central genomic and protein databases (https://https.ncbi.nlm.nih.gov/nuccore/BA000019.2/ accessed on 2 August 2022; https://www.uniprot.org/ accessed on 2 August 2022).

The specific response of *Nostoc* sp. PCC 7120 with the addition of BMAA was first investigated by Lotta Berntzon and her co-authors [104]. In their work, the authors compared the effect of BMAA and 20 standard amino acids on nitrogenase activity. With the exception of Cys, BMAA was a more potent inhibitor than all the other amino acids tested. The authors suggested that BMAA inhibited nitrogenase activity in cyanobacteria not as a potential source of nitrogen but through a mechanism affecting the metabolism of glutamate and glutamine [104]. 

Glutamate is an acceptor of ammonium ions formed by the action of the enzyme nitrogenase in heterocysts of cyanobacteria. As a result of this reaction, glutamine synthesis occurs. Glutamine is then exported from heterocysts to vegetative cells [164]. In vegetative cells, glutamine serves as a precursor to glutamate and all other amino acids. It is possible that exposure to BMAA reduces the level of glutamine and stimulates the release of glutamate from cyanobacterial cells as was observed in animal cells [163]. In this case, this will lead to rapid intracellular accumulation of ammonium (NH_4_^+^) with subsequent inhibition of nitrogen fixation [168] as observed in *Nostoc* sp. PCC 7120 [104].

Thus, this experimental study [104] demonstrated that, out of 20 proteinogenic amino acids, only Cys had a similar ability to inhibit nitrogenase activity, as did BMAA. Interestingly, Cys has been reported to have toxic effects similar to those of BMAA and to increase in toxicity in the presence of bicarbonate [169]. DAB, a neurotoxic structural isomer of BMAA [170], also has similar (though somewhat smaller) reducing effects on nitrogenase activity of *Nostoc* sp. PCC 7120 [104]. Hence, BMAA, DAB, and Cys, which are known to have a toxic effect on eukaryotic cells, are effective inhibitors of nitrogenase activity in prokaryotic cyanobacteria. The adverse effect of Cys on the activity of cyanobacterial nitrogenase was observed earlier [171]. It is of interest to determine whether Cys and BMAA (DAB) use the same mechanisms of toxicity in cyanobacteria.

Then, the regulatory role of BMAA in the development and nitrogen metabolism of diazotrophic cyanobacteria was studied using transcriptomic and proteomic experimental approaches [105,106,107,108,109]. It was found that BMAA noticeably regulates the transcription of genes specific to heterocyst and nitrogenase in diazotrophic cyanobacterium *Nostoc* (*Anabaena*) sp. PCC 7120 [105,106]. Moreover, in both of these studies, it was shown that the addition of glutamate eliminates the regulatory effect of BMAA. That is consistent with the opinion that BMAA and its carbamate are of glutamate receptors [172].

The proteomic approach has been applied to study the molecular mechanisms underlying the action of BMAA on *Nostoc* sp. PCC 7120 cells under different growth conditions [107,108,109]. Proteomics allows us to find more details that can explain why the regulatory effect of BMAA on the cellular proteome of *Nostoc* differs in conditions of growth with a lack of nitrogen and in conditions of growth with an excess of nitrogen [107,108]. 

**Figure 5:**
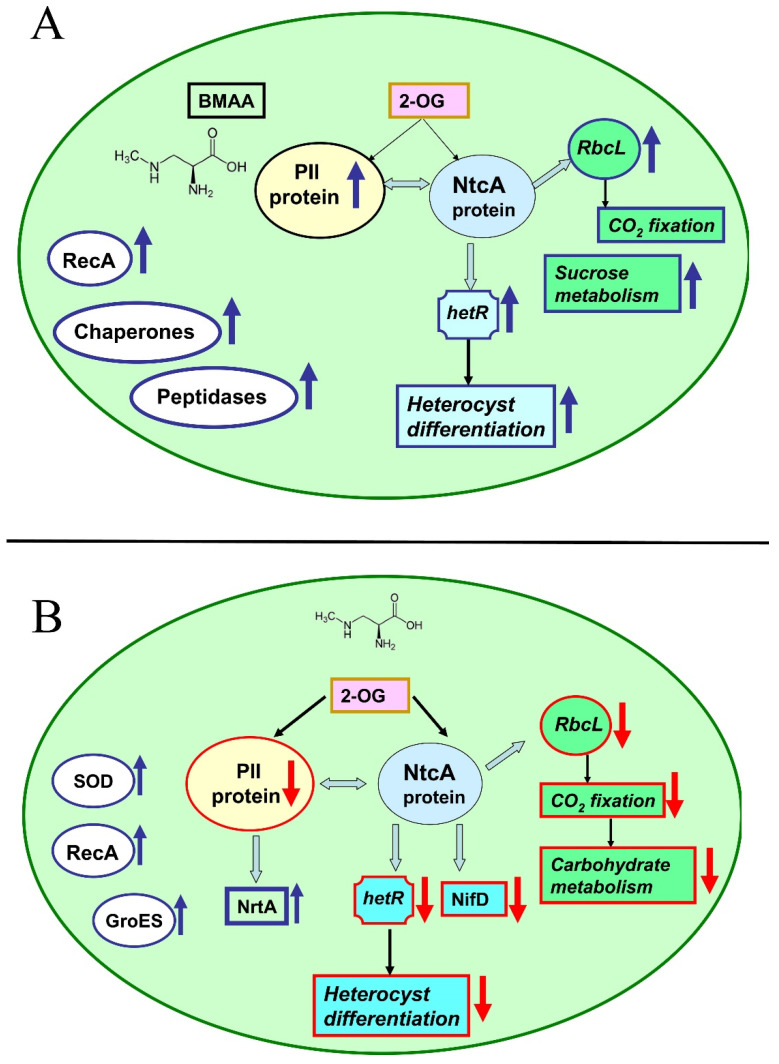
The effect of BMAA on various proteins in the cells of cyanobacteria *Nostoc* sp. PCC 7120 in nitrogen-replete conditions (**A**) and under conditions of nitrogen starvation (**B**). The light-blue arrows represent interactions between regulatory proteins PII and NicA and their main protein partners [173]. The red arrows indicate the downregulation of proteins and processes (↓), and the blue arrows indicate the upregulation of proteins and processes (↑) (modified figure from [107,108]).

The main significant difference between the effect of BMAA on cyanobacterial cells under different growth conditions is in the direction of its action on the most important regulatory protein PII [173]. BMAA down-regulates PII protein in conditions of nitrogen deficiency [107] and up-regulates PII in nitrogen-replete conditions [108] (Figure 5). This may be the main reason for the specific regulatory effect of BMAA on the expression of genes controlling the formation of heterocysts and nitrogenase activity in *Nostoc* sp. PCC 7120, which was previously detected by RT-PCR and fluorescence microscopy [105,106]. 

Since all metabolic processes in cyanobacteria cells are interconnected and well-balanced, nitrogen deficiency leads to changes in carbon metabolism and photosynthesis. Significant suppression of proteins involved in CO_2_ fixation and the photosystem I (PSI) reaction center was found [107]. The addition of BMAA leads to disruption of the synthesis of amino acids and purines, as well as DNA transcription and protein translation. Finally, many oxidative stress enzymes, chaperones, and SOS response proteins are activated under such stressful conditions [107,108].

In mature heterocysts of Nostoc sp. PCC 7120, BMAA strongly and specifically inhibits the activity of the enzyme nitrogenase [104,105] as well as the expression of the nifH gene [105] (Figure 4). This gene encodes nitrogenase reductase, one of the main components of nitrogenase. New proteomic data [109] demonstrated that BMAA downregulates proteins involved in nitrogen fixation and nitrogen metabolism. 

This cyanotoxin significantly inhibits the alpha (NifD) and beta (NifK) subunits of the molybdenum iron protein (component I) of nitrogenase. The corresponding genes (nifD and nifK) are under the transcriptional control of the global nitrogen regulator NtcA. In total, BMAA affects the expression of twenty-two proteins, which are under the transcriptional control of NtcA [109]. 

The list of these proteins includes structural proteins of nitrogenases, ABC transporters, the main components of photosystem II and ATPase, amino acid metabolism, carbohydrate metabolism enzymes, and peroxiredoxins. The imbalance of energy and the amount of metabolites leads to severe intracellular stress, which causes the activation of the starvation-induced DNA-binding protein and nine enzymes of the stress response—four proteases and four enzymes of the SOS response and DNA repair [109] (Figure 5).

**Figure 6:**
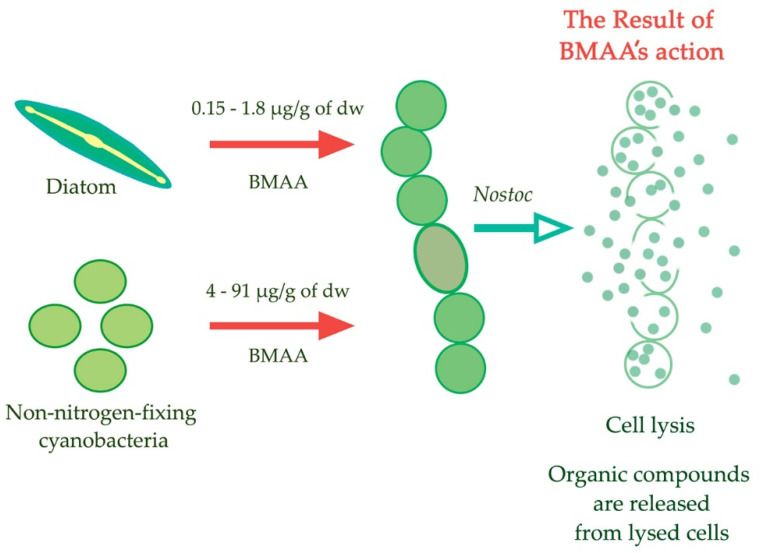
Illustration of the “allelopathic tool” hypothesis. BMAA can be used in the fight for organic nitrogen [109].

A reasonable question arises as to why cyanobacteria and other microalgae synthesize BMAA? It was assumed [106,109] that BMAA can be used by toxin-producing phytoplankton (cyanobacteria, diatoms, and dinoflagellates) as a possible allelopathic tool for controlling populations of cyanobacterial cells competing for nitrogen and other resources (Figure 6). This assumption is consistent with the observations of Downing et al. that non-diazotrophic unicellular cyanobacteria begin to synthesize BMAA in nitrogen-limited conditions [27]. 

It was shown [104,159] that cyanobacteria Synechocystis sp. PCC 6803 and Anabaena (Nostoc) sp. PCC 7120 rapidly absorbed exogenous BMAA in proportion to its concentration. In a recent study [111], the authors suggested that BMAA is taken up mainly through N-I and N-II amino acid transport systems in Anabaena sp. PCC 7120. In this work, UPLC-MS/MS showed that a double mutant, in which both transport systems were disrupted, could not absorb exogenous BMAA. However, there were no results of studying the individual uptake of BMAA by mutants carrying mutations in each of the N-I and N-II systems, and the effects for a single mutant ∆natl that had a violation in the N-III transport system. Note that the mutant ∆natl was also BMAA resistant [111]. Thus, additional studies are needed to clarify the features of BMAA transport in cyanobacteria cells. More research is also needed to understand the functional role(s) of BMAA in the microalgae community as well.

### 4.6. Can BMAA Induce Oxidative Stress and Apoptosis?

Proteinogenic amino acids form Schiff bases at an intermediate stage of transamination, while the pyridoxal-5′ -phosphate molecule acts as a coenzyme in mammals [174]. BMAA likely reacts similarly with pyridoxal-5′-phosphate in a neutral medium and forms a Schiff base. As a result of the reaction of BMAA with pyridoxal-5’-phosphate, methylamine and ammonia are formed as end products at pH 7.0 and 37 °C [162]. Then, methylamine is oxidized to formaldehyde, hydrogen peroxide, and ammonia. This oxidation is catalyzed by a semicarbazide-sensitive amine oxidase [89]. The semicarbazide-sensitive amine oxidase catalyzes reactions involving aliphatic and aromatic monoamines and is classified as a monoamine oxidase [123]. This enzyme is present in the blood and vascular system, including the circulatory system of the human brain [175,176]. The enzyme has also been found in plants [123,177]. Therefore, in the presence of this enzyme, BMAA can react to form reactive oxygen species (ROS), such as hydrogen peroxide, formaldehyde, and ammonia, which are toxic to cells.

BMAA inhibits the cystine/glutamate antiporter system and, thus, reduces the intracellular concentration of glutathione and causes oxidative stress [112]. The concentration of reactive oxygen species and protein oxidation increased at high concentrations of BMAA (≥1 mM, 48-hour exposure). A significant increase in the activity of caspases 3 and 7, which control chromatin condensation during apoptosis, was observed at BMAA concentrations ≥2 mM [87]. 

The process of protein ubiquitination (protein degradation signal) was enhanced at low, non-excitotoxic BMAA concentrations (≥0.1 mM, 48-hour exposure), while the activity of the 20S proteasome and caspase 12 increased [176]. BMAA promotes oxidative stress and a significant increase in the formation of ROS in animal cells [122,124,125,148,178] and apoptosis [179]. Moreover, exposure to BMAA reduces DNA methylation in cells, which means that the effect of BMAA may be associated with epigenetic changes in cells [134].

The addition of exogenous BMAA leads to upregulation of the main stress response proteins in cyanobacterium *Nostoc* sp. PCC 7120 [107,108,109]. During nitrogen starvation, BMAA upregulates peroxiredoxin (*alr4641*), superoxide dismutase (*all0070*), glutathione reductase (*all4968*), and thioredoxin reductase (*all0737*) in cyanobacteria cells [107]. BMAA treatment induces nine enzymes involved in the stress response in diazotrophic *Nostoc* sp. PCC 7120 [109]. In all three different growth conditions (diazotrophic growth, conditions with excess nitrogen, and nitrogen-starvation), BMAA also causes highly upregulation of the RecA protein involved in DNA repair [107,108,109] (Figure 5).

Exogenous BMAA demonstrates different effects on oxidative stress enzymes depending on the BMAA concentration, exposure time, and plant species. This cyanotoxin inhibits or induces oxidative stress enzymes [72,176]. Antioxidant responses towards BMAA exposure are different in different aquatic plant species. In [72], the effect of BMAA on macrophyte responses to oxidative stress in Ceratophyllum demersum (known as hornwort) was studied. BMAA had an inhibitory effect on all the oxidative stress response enzymes—superoxide dismutase, catalase, guaiacol peroxidase, glutathione peroxidase, and glutathione reductase in this plant [72]. In another study [25], the peroxidase activity for three species of mosses (Riccia fluitans, Lomariopsis lineata, and Taxiphyllum barbieri) showed an increase in enzymatic activity at an exposure concentration of 10 mg L^−1^ BMAA. However, exposure to 100 mg L^−1^ resulted in inhibition of peroxidase activity. The activity of enzymes changed during time. The L. lineata treated with both concentrations of BMAA (10 mg L^−1^ and 100 mg L^−1^) showed increased peroxidase activity throughout the experiment and increased glutathione S-transferase activity after a longer exposure for two weeks [25].

Plants are considered a “Green Liver System” that can remove the cyanobacterial toxin BMAA from water without severe physiological damage [25]. Aquatic fungi can also perform the biotransformation of cyanotoxins and demonstrate increased resistance to oxidative stress. BMAA inhibits only glutathione reductase in Mucor hiemalis fungi; however, catalase, glutathione peroxidase, and glutathione S-transferase remained unaffected [25].

## 5. The Biological Significance of BMAA and Further Research Perspectives

The ability of various species of cyanobacteria, microalgae, and some plants to synthesize BMAA in a wide range of concentrations—from nanograms to thousands of micrograms per gram of dry weight—may indicate the biological significance of the role that this molecule plays in their lives. The accumulated data indicate the regulatory role of BMAA in the physiology of plants and algae. The strong specific effect of BMAA on nitrogen fixation and cell differentiation in diazotrophic cyanobacteria allows us to assume that this compound has some allelopathic functions and can be used by producers in conditions of nitrogen starvation [104,105,106,107,108,109] (Figure 6).

A new question arises regarding the possible role of BMAA in the symbiosis of plants and cyanobacteria. Both partners can likely use this molecule “to sort things out“ to regulate the metabolism of carbon and nitrogen in the cells of both symbionts. The discovery of GluR-like genes in *C. rumphii* raises an intriguing question about the possibility that endogenous BMAA may interact with native cycad GluRs as a regulatory molecule [46]. Future studies may help to understand the role of many GluRs in plants and the role of BMAA in endogenous plant signaling and protection from herbivores. Information on the functionality of glutamate receptors of plants and cyanobacteria is also scarce [152,156]. Cox et al. [9] suggested that BMAA may serve as an antiherbivory compound in cycads due to the high concentration of BMAA in the developing reproductive organs of *C. micronesica* (1546 μg/g of BMAA in immature male sporangia and 1161 μg/g of BMAA in the outer integument layer of cycas seed sarcotesta) (Table 1). This assumption requires experimental verification.

Non-protein amino acids and BMAA, in particular, are secondary metabolites. Secondary metabolites are regulatory molecules that control certain metabolic processes in the cells of various organisms. For example, these molecules provide the function of bacterial communication in microbial communities and have a distinct allelopathic effect on competition between microorganisms [180,181,182]. Little is known about the exchange of metabolites in the symbiosis of cyanobacteria with feathery mosses. The epiphytic symbiosis of moss and cyanobacteria differs from other symbioses of cyanobacteria and plants [183]. Metabolic relationships in another type of symbioses—symbioses of diatoms and cyanobacteria—are also insufficiently studied. Both partners in such a symbiosis are potential BMAA producers [17,31], and their metabolism is vulnerable to the action of this amino acid in micromolar concentrations [36,103,104,105,106,107,108,109,110,111,184]. 

It was recently demonstrated that BMAA (1 μM) affects the growth of diatom *P. tricornutum* by increasing oxidative stress and reducing carbon fixation [184]. Three models of metabolite exchange between heterocyst-forming cyanobacteria as symbionts and diatoms as hosts were recently proposed [185]. How can BMAA be involved (if at all) in the processes of metabolite exchange in such a symbiosis? This field of experimental research is fascinating and promising.

Of great interest is the presence of BMAA as a molecular element of antimicrobial peptides [14] in the widespread bacteria *Paenibacillus* spp. [186]. This makes it possible to explore another side of the involvement of BMAA in biological molecules and opens up new areas of research. Antimicrobial peptides [187] are multifunctional biological molecules that play an important regulatory roles in bacterial Quorum Sensing, apoptosis, and other biological processes.

Cyanobacteria synthesize a large number of secondary metabolites, and these metabolites can act together as instruments of allelopathy [182]. Further studies of the functional activity of BMAA in a mixture with other cyanotoxins [188] may help in a broad understanding of its biological role.

Another area of research should focus on developing new, more accurate methods for detecting BMAA in various samples. The use of advanced analytical methods in combination with methods of functional proteomics and metabolomics will deepen our knowledge of the role of BMAA in the dynamics of neurodegenerative processes [189] and its regulatory role in natural ecosystems.

## 6. Conclusions

Understanding the biological functions of toxic secondary metabolites in the life cycle of producing organisms and their effects on other organisms is a fundamental task of environmental microbiology and toxicology. This review summarizes, for the first time, the current knowledge regarding the significant regulatory effects of diaminoacid BMAA for its main producers—cyanobacteria. We demonstrated that proteomic and other “omic” methods reveal the complex regulatory effect of BMAA on all major metabolic pathways of cyanobacteria cells. 

The review highlights some unsolved problems of BMAA biology: (i) the unknown role of BMAA in natural systems, such as cyanobacterial biofilms, as well as in many symbioses that cyanobacteria form with other organisms (microalgae, plants, animals, and humans); (ii) the functional significance of BMAA in peptides and its binding to proteins; (iii) the microbiome-based monitoring of BMAA in animal and human intestines (which can be a sensitive and comprehensive tool for rapid health risk assessment); and (iv) the lack of comprehensive studies of mutants of cyanobacteria, plants, and microalgae resistant to the action of BMAA. Interdisciplinary approaches, including genetics, transcriptomics, proteomics, and metabolomics, will help researchers to solve these problems.

## Figures and Tables

**Table 1:** BMAA has been detected in a range of organisms (some examples are presented).

Source	Concentration of BMAA μg/g Dry Weight of Sample	Method of Identification	Reference
Cyanobacteria *Nostoc*	0.3	HPLC-MS	[9]
Cycad/cyanobacterial symbiosis	37–1161	HPLC-MS	[9]
the immature male sporangi of *Cycas micronesica*	1546	HPLC-MS	[9]
the outer integument layer of the cycas seed sarcotesta	1161	HPLC-MS	[9]
Flying Foxes	3556	HPLC-MS	[9]
brain tissues from the frontal cortex of six Chamorro patients	6	HPLC-MS	[9]
brain tissues from the frontal cortex of two Alzheimer’s patients from Canada	6.6	HPLC-MS	[9]
*Azolla filiculoides* with cyanobacterial symbionts in its leaves	2	HPLC-MS	[9]
*Gunnera kauaiensi* with cyanobacterial symbionts in its leaves	4	HPLC-MS	[9]
*Nostoc* PCC 9305 (symbiont of *Anthoceros* )	156 (free) 1400 (protein-bound)	HPLC-MS	[17]
*Nostoc* PCC 7422 (symbiont of *Cycas*)	962 (protein-bound)	HPLC-MS	[17]
*Nostoc* 8001 (symbiont of *Gunnera monoica*)	203 (free) 664 (protein-bound)	HPLC-MS	[17]
*Microcystis* PCC 7820 (Freshwater, Scotland)	6 (free) 12 (protein-bound)	HPLC-MS	[17]
*Chroococcidiopsis indica* GQ2-7 (Marine coral)	435 (free) 76 (protein-bound)	HPLC-MS	[17]
*Chroococcidiopsis indica* GT-3-26 (Marine rock)	1306 (free) 5415 (protein-bound)	HPLC-MS	[17]
*Cylindrospermopsis raciborskii* CR3 (Freshwater, Australia)	6478 (free) 14 (protein-bound)	HPLC-MS	[17]
*Nostoc* 268 (Brackish Water, Baltic Sea)	34 (free) 274 (protein-bound)	HPLC-MS	[17]
*Nostoc* sp. CMMED 01 (Marine, Hawaiian Islands)	1243 (free) 1070 (protein-bound)	HPLC-MS	[17]
*Chlorogloeopsis* PCC 6912 (Soil, India)	758 (free)	HPLC-MS	[17]
*Fischerella* PCC 7521 (Yellowstone, hot spring, USA)	44 (free) 175 (protein-bound)	HPLC-MS	[17]
*Scytonema* PCC 7110 (Limestone cave, Bermuda)	1733 (protein-bound)	HPLC-MS	[17]
*Mytilus edulis* (common mussel, Sweden’s west coast)	0.15–0.2	HPLC-MS/MS	[23]
*Ostrea edulis* (common oyster, Sweden’s west coast )	0.006–0.14	HPLC-MS/MS	[23]
*Scophthalmus maximus* (turbot) (Brain tissue)	0.047–1.29	HPLC-MS/MS	[23]
*Microcystis* PCC7806 and *Synechocystis* J341	the relative abundance of labeled amino acids based on LC/MS/MS peak areas	stable isotope 15N and UPLC-MS/MS	[27]
*Thalassiosira* sp. (CCAP 1085/15) diatom	0.0033	UHPLC-MS/MS	[30]
*Skeletonema marinoi* isolates (SAAE 08603) diatom	0.0011	UHPLC-MS/MS	[30]
*Phaeodactylum tricornutum* diatom	0.20–1.4	HPLC-MS/MS	[31]
*Cyclotella* (diatom from Lake Liddell )	0.103 (free) 0.154 (protein-bound)	LC-MS/MS	[32]
*Navicula* (from Lostock Dam)	0.151 (free) 0.369 (protein-bound)	LC-MS/MS	[32]
*Cycas micronesica* K.D. Hill Gametophyte Southern	1.94	GC-MS	[35]
*Cycas micronesica* K.D. Hill Gametophyte Nothern	3.32	GC-MS	[35]
*Cycas micronesica* K.D. Hill total plant Southern	3.44	GC-MS	[35]
*Cycas micronesica* K.D. Hill total plant Nothern	4.9	GC-MS	[35]
Blue mussel (*Mytilus edulis*) Sweden (west coast)	0.08–0.9	UHPLC-MS/MS	[39]
Oyster (*Ostrea edulis*) Greece	0.32	UHPLC-MS/MS	[39]
Oyster (*Crassostrea gigas*) France	0.66	UHPLC-MS/MS	[39]
*Triticum aestivum* (mature seeds) via irrigation	217 ± 150 ng g FW^−1^ (protein-bound)	UHPLC-MS/MS	[43]
chicken tissues (i.e., muscle, liver, brain, and eye)	0.0045–0.03 (free) 0.03–0.23 (protein-bound)	UHPLC-MS/MS	[45]

## Data Availability

Not applicable.

## References

[1] Vega A., Bell E.A. (1967). α-Amino-β-methylaminopropionic acid, a new amino acid from seeds of Cycas circinalis. Phytochemistry.

[2] Nunn P.B. (2017). 50 years of research on α-amino-β-methylaminopropionic acid (β-methylaminoalanine). Phytochemistry.

[3] Vranova V., Rejsek K., Skene K.R., Formanek P. (2011). Non-protein amino acids: Plant, soil and ecosystem interactions. Plant Soil.

[4] Shenker M., Fan T.W.M., Crowley D.E. (2001). Phytosiderophores influence on cadmium mobilization and uptake by wheat and barley plants. J. Environ. Qual..

[5] Casagrande D.J., Given P.H. (1980). Geochemistry of amino acids in some Florida peat accumulation-II. Amino acid distributions. Geochim. Cosmochim. Acta.

[6] Walsh C.T., O’Brien R.V., Khosla C. (2013). Nonproteinogenic Amino Acid Building Blocks for Nonribosomal Peptide and Hybrid Polyketide Scaffolds. Angew. Chem. Int. Ed..

[7] Hirano A., Malamud N., Elizan T.S., Kurland L.T. (1966). Amyotrophic lateral sclerosis and Parkinsonism-dementia complex on Guam. Further pathologic studies. Arch. Neurol..

[8] Spencer P.S., Nunn P.B., Hugon J., Ludolph A.C., Ross S.M., Roy D.N., Robertson R.C. (1987). Guam amyotrophic lateral sclerosis-parkinsonism-dementia linked to a plant excitant neurotoxin. Science.

[9] Cox P.A., Banack S.A., Murch S.J. (2003). Biomagnification of cyanobacterial neurotoxins and neurodegenerative disease among the Chamorro people of Guam. Proc. Natl. Acad. Sci. USA.

[10] Murch S.J., Cox P.A., Banack S.A. (2004). A mechanism for slow release of biomagnified cyanobacterial neurotoxins and neurodegenerative disease in Guam. Proc. Natl. Acad. Sci. USA.

[11] Murch S.J., Cox P.A., Banack S.A., Steele J.C., Sacks O.W. (2004). Occurrence of beta-methylamino-l-alanine (BMAA) in ALS/PDC patients from Guam. Actaneurologica Scand..

[12] Bradley W.G., Mash D.C. (2009). Beyond Guam: The cyanobacteria/BMAA hypothesis of the cause of ALS and other neurodegenerative diseases. Amyotroph. Lateral Scler..

[13] Popova A.A., Koksharova O.A. (2016). Neurotoxic non-proteinogenic amino acid β-N-methylamino-L-alanine and its role in biological systems. Biochemistry.

[14] Nunn P.B., Codd G.A. (2019). Environmental distribution of the neurotoxin l-BMAA in *Paenibacillus* species. Toxicol. Res..

[15] Brody J.A., Stanhope J.M., Kurland L.T. (1975). Patterns of amyotrophic lateral sclerosis and parkinsonism-dementia on Guam. Contemp Neurol Ser..

[16] Pablo J., Banack S.A., Cox P.A., Johnson T.E., Papapetropoulos S., Bradley W.G., Buck A., Mash D.C. (2009). Cyanobacterial neurotoxin BMAA in ALS and Alzheimer’s disease. Acta Neurol Scand..

[17] Cox P.A., Banack S.A., Murch S.J., Rasmussen U., Tien G., Bidigare R.R., Metcalf J.S., Morrison L.F., Codd G.A., Bergman B. (2005). Diverse taxa of cyanobacteria produce beta-N-methylamino-L-alanine, a neurotoxic amino acid. Proc. Natl. Acad. Sci. USA.

[18] Esterhuizen M., Downing T.G. (2008). β-N-methylamino-L-alanine (BMAA) in novel South African cyanobacterial isolates. Ecotoxicol. Environ. Saf..

[19] Banack S.A., Johnson H.E., Cheng R., Cox P.A. (2007). Production of the neurotoxin BMAA by a marine cyanobacterium. Mar. Drugs.

[20] Johnson H.E., King S.R., Banack S.A., Webster C., Callanaupa W.J., Cox P.A. (2008). Cyanobacteria (*Nostoc commune*) used as a dietary item in the Peruvian highlands produce the neurotoxic amino acid BMAA. J. Ethnopharmacol..

[21] Metcalf J.S., Banack S.A., Lindsay J., Morrison L.F., Cox P.A., Codd G.A. (2008). Co-occurrence of beta-N-methylamino-L-alanine, a neurotoxic amino acid with other cyanobacterial toxins in British waterbodies, 1990–2004. Environ. Microbiol..

[22] Brand L.E., Pablo J., Compton A., Hammerschlag N., Mash D.C. (2010). Cyanobacterial blooms and the occurrence of the neurotoxin beta-N-methylamino-L-alanine (BMAA) in South Florida aquatic food webs. Harmful Algae.

[23] Jonasson S., Eriksson J., Berntzon L., Spacil Z., Ilag L.L., Ronnevi L.O., Rasmussen U., Bergman B. (2010). Transfer of a cyanobacterial neurotoxin within a temperate aquatic ecosystem suggests pathways for human exposure. Proc. Natl. Acad. Sci. USA.

[24] Cervantes Cianca R.C., Baptista M.S., Lopes V.R., Vasconcelos V.M. (2012). The non-protein amino acid β-N-methylamino-L-alanine in Portuguese cyanobacterial isolates. Amino Acids.

[25] Contardo-Jara V., Sebastian Funke M., Peuthert A., Pflugmacher S. (2013). β-N-Methylamino-L-alanine exposure alters defense against oxidative stress in aquatic plants *Lomariopsis lineata*, *Fontinalis antipyretica*, *Riccia fluitans* and *Taxiphyllum barbieri*. Ecotoxicol. Environ. Saf..

[26] Spacil Z., Eriksson J., Jonasson S., Rasmussen U., Ilag L.L., Bergman B. (2009). Analytical protocol for identification of BMAA and DAB in biological samples. Analyst.

[27] Downing S., Banack S.A., Metcalf J.S., Cox P.A., Downing T.G. (2011). Nitrogen starvation of cyanobacteria results in the production of β-N-methylamino-L-alanine. Toxicon.

[28] Violi J.P., Mitrovic S.M., Colville A., Main B.J., Rodgers K.J. (2019). Prevalence of β-methylamino-L-alanine (BMAA) and its isomers in freshwater cyanobacteria isolated from eastern Australia. Ecotoxicol Environ Saf..

[29] Jungblut A.D., Wilbraham J., Banack S.A., Metcalf J.S., Codd G.A. (2018). Microcystins, BMAA and BMAA isomers in 100-year-old Antarctic cyanobacterial mats collected during Captain R.F. Scott’s Discovery Expedition. Eur. J. Phycol..

[30] Jiang L., Eriksson J., Lage S., Jonasson S., Shams S., Mehine M., Ilag L.L., Rasmussen U. (2014). Diatoms: A novel source for the neurotoxin BMAA in aquatic environments. PLoS ONE.

[31] Réveillon D., Séchet V., Hess P., Amzil Z. (2016). Production of BMAA and DAB by diatoms (*Phaeodactylum tricornutum*, *Chaetoceros* sp., *Chaetoceros calcitrans* and, *Thalassiosira pseudonana*) and bacteria isolated from a diatom culture. Harmful Algae.

[32] Violi J.P., Facey J.A., Mitrovic S.M., Colville A., Rodgers K.J. (2019). Production of β-methylamino-L-alanine (BMAA) and Its Isomers by Freshwater Diatoms. Toxins.

[33] Bates S.S., Lundholm N., Hubbard K.A., Montresor M., Leaw C.P., Seckbach J., Gordon R. (2019). Toxic and harmful marine diatoms. Diatoms: Fundamentals and Applications.

[34] Lage S., Costa P.R., Moita T., Eriksson J., Rasmussen U., Rydberg S.J. (2014). BMAA in shellfish from two Portuguese transitional water bodies suggests the marine dinoflagellate Gymnodinium catenatum as a potential BMAA source. Aquat. Toxicol..

[35] Marler T.E., Snyder L.R., Shaw C.A. (2010). *Cycas micronesica* (Cycadales) plants devoid of endophytic cyanobacteria increase in β-methylamino-L-alanine. Toxicon.

[36] Downing T.G., Phelan R.R., Downing S. (2015). A potential physiological role for cyanotoxins in cyanobacteria of arid environments. J. Arid Environ..

[37] Downing S., Downing T.G. (2016). The metabolism of the non proteinogenic amino acid β-N-methylamino-L-alanine (BMAA) in the cyanobacterium *Synechocystis* PCC 6803. Toxicon.

[38] Nunn P.B., Codd G.A. (2017). Metabolic solutions to the biosynthesis of some diaminomonocarboxylic acids in nature: Formation in cyanobacteria of the neurotoxins 3-N-methyl-2,3-diaminopropanoic acid (BMAA) and 2,4-diaminobutanoic acid (2,4-DAB). Phytochemistry.

[39] Jiang L., Kiselova N., Rosén J., Ilag L.L. (2014). Quantification of neurotoxin BMAA (β-N-methylamino-L-alanine) in seafood from Swedish markets. Sci. Rep..

[40] Salomonsson M.L., Fredriksson E., Alfjorden A., Hedeland M., Bondesson U. (2015). Seafood sold in Sweden contains BMAA: A study of free and total concentrations with UHPLC-MS/MS and dansyl chloride derivatization. Toxicol. Rep..

[41] Hammerschlag N., Davis D.A., Mondo K., Seely M.S., Murch S.J., Glover W.B., Divoll T., Evers D.C., Mash D.C. (2016). Cyanobacterial Neurotoxin BMAA and Mercury in Sharks. Toxins.

[42] Regueiro J., Negreira N., Carreira-Casais A., Pérez-Lamela C., Simal-Gándara J. (2017). Dietary exposure and neurotoxicity of the environmental free and bound toxin β-N-methylamino-l-alanine. Food Res. Int..

[43] Contardo-Jara V., Schwanemann T., Esterhuizen-Londt M., Pflugmacher S. (2018). Protein association of β-N-methylamino-L-alanine in *Triticum aestivum* via irrigation. Food Addit. Contam. Part A.

[44] Esterhuizen-Londt M., Pflugmacher S. (2019). Vegetables cultivated with exposure to pure and naturally occurring β-N-methylamino-L-alanine (BMAA) via irrigation. Environ. Res..

[45] Kim S.-Y., Rydberg S. (2020). Transfer of the Neurotoxin β-N-methylamino-l-alanine (BMAA) in the Agro–Aqua Cycle. Mar. Drugs.

[46] Brenner E.D., Stevenson D.W., McCombie R.W., Katari M., A Rudd S., Mayer K.F.X., Palenchar P.M., Runko S.J., Twigg R.W., Dai G. (2003). Expressed sequence tag analysis in Cycas, the most primitive living seed plant. Genome Biol..

[47] Mantas M.J.Q., Nunn P.B., Codd G.A., Barker D. (2022). Genomic insights into the biosynthesis and physiology of the cyanobacterial neurotoxin 3-N-methyl-2,3-diaminopropanoic acid (BMAA). Phytochemistry.

[48] Müller S., Garcia-Gonzalez E., Mainz A., Hertlein G., Heid N.C., Mösker E., van den Elst H.V., Overkleeft H.S., Genersch E., Süssmuth R.D. (2014). Paenilamicin: Structure and biosynthesis of a hybrid nonribosomal peptide/polyketide antibiotic from the bee pathogen *Paenibacillus larvae*. Angew. Chem. Int. Ed..

[49] Du X., Liu H., Yuan L., Wang Y., Ma Y., Wang R., Chen X., Losiewicz M., Guo H., Zhang H. (2019). The Diversity of Cyanobacterial Toxins on Structural Characterization, Distribution and Identification: A Systematic Review. Toxins.

[50] Berstad K., Berstad J.E.R. (2017). Parkinson’s disease; the hibernating spore hypothesis. Med. Hypotheses.

[51] Brenner S. (2013). Blue-green algae or cyanobacteria in the intestinal micro-flora may produce neurotoxins such as β-N-Methylamino-L-Alanine (BMAA) which may be related to development of amyotrophic lateral sclerosis, Alzheimer’s disease and Parkinson-Dementia-Complex in humans and equine motor neuron disease in horses. Med. Hypotheses.

[52] Nunes-Costa D., Magalhães J.D., G-Fernandes M., Cardoso S.M., Empadinhas N. (2020). Microbial BMAA and the Pathway for Parkinson’s Disease Neurodegeneration. Front. Aging Neurosci..

[53] Di Rienzi S.C., Sharon I., Wrighton K.C., Koren O., Hug L.A., Thomas B.C., Goodrich J.K., Bell J., Spector T.D., Banfield J.T. (2013). The human gut and groundwater harbor non-photosynthetic bacteria belonging to a new candidate phylum sibling to Cyanobacteria. Elife.

[54] Soo R.M., Skennerton C.T., Sekiguchi Y., Imelfort M., Paech S.J., Dennis P.G., Steen J.A., Parks D.H., Tyson G.W., Hugenholtz P. (2014). An expanded genomic representation of the phylum cyanobacteria. Genome. Biol. Evol..

[55] Rosen J., Hellenas K.E. (2008). Determination of the neurotoxin BMAA (beta-N-methylamino-L-alanine) in cycad seed and cyanobacteria by LC-MS/MS (liquid chromatography tandem mass spectrometry). Analyst.

[56] Bishop S.L., Murch S.J. (2020). A systematic review of analytical methods for the detection and quantification of β-N-methylamino-l-alanine (BMAA). Analyst.

[57] Faassen E.J. (2014). Presence of the neurotoxin BMAA in aquatic ecosystems: What do we really know?. Toxins.

[58] Faassen E., Antoniou M., Beekman-Lukassen W., Bláhová L., Chernova E., Christophoridis C., Combes A., Edwards C., Fastner J., Harmsen J. (2016). A Collaborative Evaluation of LC-MS/MS Based Methods for BMAA Analysis: Soluble Bound BMAA Found to Be an Important Fraction. Mar. Drugs.

[59] Porojan C., Mitrovic S.M., Yeo D.C.J., Furey A. (2016). Overview of the potent cyanobacterial neurotoxin β-methylamino-L-alanine (BMAA) and its analytical determination. Food Addit. Contam. Part A.

[60] Dunlop R.A., Banack S.A., Bishop S.L., Metcalf J.S., Murch S.J., Davis D.A., Stommel E.W., Karlsson O., Brittebo E.B., Chatziefthimiou A.D. (2021). Is exposure to BMAA a risk factor for neurodegenerative diseases? A response to a critical review of the BMAA hypothesis. Neurotox. Res..

[61] Vo Duy S., Munoz G., Dinh Q.T., Tien Do D., Simon D.F., Sauveґ S. (2019). Analysis of the neurotoxin β-N-methylamino-L-alanine (BMAA) and isomers in surface water by FMOC derivatization liquid chromatography high resolution mass spectrometry. PLoS ONE.

[62] Banack S.A. (2020). Second laboratory validation of β-N-methylamino-L-alanine, N-(2aminoethyl)glycine, and 2,4-diaminobuytric acid by ultra-performance liquid chromatography and tandem mass spectrometry. Neurotox. Res..

[63] Jiang L., Johnston E., Aberg K.M., Nilsson U., Ilag L.L. (2013). Strategy for quantifying trace levels of BMAA in cyanobacteria by LC/MS/MS. Anal. Bioanal. Chem..

[64] Pan M., Mabry T.J., Cao P., Moini M. (1997). Identification of nonprotein amino acids from cycad seeds as N-ethoxycarbonyl ethyl ester derivatives by positive chemical-ionization gas chromatography-mass spectrometry. J. Chromatogr. A.

[65] Guo T., Geis S., Hedman C., Arndt M., Krick W., Sonzogni W. (2007). Characterization of ethyl chloroformate derivative of beta-methylamino-L-alanine. J. Am. Soc. Mass. Spectr..

[66] Baptista M.S., Cianca R.C., Lopes V.R., Almeida C.M., Vasconcelos V.M. (2011). Determination of the nonprotein amino acid beta-N-methylamino-L-alanine in estuarine cyanobacteria by capillary electrophoresis. Toxicon.

[67] Beach D.G., Kerrin E.S., Quilliam M.A. (2015). Selective quantitation of the neurotoxin BMAA by use of hydrophilic-interaction liquid chromatography-differential mobility spectrometry-tandem mass spectrometry (HILIC-DMS-MS/MS). Anal. Bioanal. Chem..

[68] Beach D.G., Kerrin E.S., Giddings S.D., Quilliam M.A., McCarron P. (2018). Differential mobility-mass spectrometry double spike isotope dilution study of release of beta-methylaminoalanine and proteinogenic amino acids during biological sample hydrolysis. Sci. Rep..

[69] Moura S., De Almeida Ultramari M., Mendes Louzada de Paula D., Yonamine M., Pinto E. (2009). 1H NMR determination of β-N-methylamino-l-alanine (l-BMAA) in environmental and biological samples. Toxicon.

[70] Cohen S.A. (2012). Analytical techniques for the detection of α-amino-β-methylaminopropionic acid. Analyst.

[71] Masseret E., Banack S.A., Boumediene F., Abadie E., Brient L., Pernet F., Juntas-Morales R., Pageot N., Metcalf J., Cox P. (2013). Detection of BMAA in the marine environment of an ALS cluster in Southern France. PLoS ONE.

[72] Esterhuizen-Londt M., Pflugmacher S., Downing T.G. (2011). The effect of β-N-methylamino-L-alanine (BMAA) on oxidative stress response enzymes of the macrophyte *Ceratophyllum demersum*. Toxicon.

[73] Salomonsson M., Hansson A., Bondesson U. (2013). Development and in-house validation of a method for quantification of BMAA in mussels using dansyl chloride derivatization and ultra-performance liquid chromatography tandem mass spectrometry. Anal. Methods.

[74] Downing S., Contardo-Jara V., Pflugmacher S., Downing T.G. (2014). The fate of the cyanobacterial toxin β-N-methylamino-L-alanine in freshwater mussels. Ecotoxicol. Environ. Saf..

[75] Zurita J., Zguna N., Andrýs R., Strzelczak A., Jiang L., Thorsen G., Ilag L.L. (2019). Chiral analysis of β-methylamino alanine (BMAA) enantiomers after (+)-1-(9-fluorenyl)-ethyl chloroformate (FLEC) derivatization and LC-MS/MS. Anal. Methods.

[76] Banack S.A., Metcalf J.S., Spacil Z., Downing T.G., Downing S., Long A., Nunn P.B., Cox P.A. (2011). Distinguishing the cyanobacterial neurotoxin beta-N-methylamino-L-alanine (BMAA) from other diamino acids. Toxicon.

[77] Lage S., Burian A., Rasmussen U., Costa P.R., Annadotter H., Godhe A., Rydberg S. (2016). BMAA extraction of cyanobacteria samples: Which method to choose?. Environ. Sci. Pollut. Res. Int..

[78] Lobner D., Piana P.M.T., Salous A.K., Peoples R.W. (2007). β-N-methylamino-l-alanine enhances neurotoxicity through multiple mechanisms. Neurobiol. Dis..

[79] Davis D.A., Cox P.A., Banack S.A., Lecusay P.D., Garamszegi S.P., Hagan M.J., Powell J.T., Metcalf J.S., Palmour R.M., Beierschmitt A. (2020). L-serine reduces spinal cord pathology in a vervet model of preclinical ALS/MND. J. Neuropathol. Exp. Neurol..

[80] Weiss J.H., Christine C.W., Choi D.W. (1989). Bicarbonate dependence of glutamate receptor activation by beta-N-methylamino-L-alanine: Channel recording and study with related compounds. Neuron.

[81] Weiss J.H., Koh J.-Y., Choi D.W. (1989). Neurotoxicity of β-N-methylamino-L-alanine (BMAA) and β-N-oxalylamino-L-alanine (BOAA) on cultured cortical neurons. Brain Res..

[82] Seawright A.A., Brown A.W., Nolan C.C., Cavanagh J.B. (1990). Selective degeneration of cerebellar cortical neurons caused by cycad neurotoxin, L-β-methylaminoalanine (L-BMAA), in rats. Neuropathol. Appl. Neurobiol..

[83] Purdie E.L., Samsudin S., Eddy F.B., Codd G.A. (2009). Effects of the cyanobacterial neurotoxin β-N-methylamino-L-alanine on the early-life stage development of zebrafish (*Danio rerio*). Aquat. Toxicol..

[84] Purdie E.L., Metcalf J.S., Kashmiri S., Codd G.A. (2009). Toxicity of the cyanobacterial neurotoxin β -N-methylamino-L-alanine to three aquatic animal species. Amyotroph. Lateral Scler..

[85] Zhou X., Escala W., Papapetropoulos S., Bradley W.G., Zhai R.G. (2009). BMAA neurotoxicity in Drosophila. Amyotroph Lateral Scler..

[86] Karlsson O., Berg A.L., Lindstrom A.K., Hanrieder J., Arnerup G., Roman E., Bergquist J., Lindquist N.G., Brittebo E.B., Andersson M. (2012). Neonatal exposure to the cyanobacterial toxin BMAA induces changes in protein expression, and neurodegeneration in adult hippocampus. Toxicol. Sci..

[87] Yin H.Z., Yu S., Hsu C.-I., Liu J., Acab A., Wu R., Tao A., Chiang B.J., Weiss J.H. (2014). Intrathecal infusion of BMAA induces selective motor neuron damage and astrogliosis in the ventral horn of the spinal cord. Exp. Neurol..

[88] Wang S., Qiu J., Zhao M., Li F., Yu R., Li A. (2020). Accumulation and distribution of neurotoxin BMAA in aquatic animals and effect on the behavior of zebrafish in a T-maze test. Toxicon.

[89] Nunn P.B. (2009). Three phases of research on β-N-methylamino-L-alanine (BMAA)—A neurotoxic amino acid. Amyotroph. Lateral Scler..

[90] De Munck E., Muñoz-Sáez E., Miguel B.G., Solas M.T., Martínez A., Arahuetes R.M. (2013). β-N-methylamino-l-alanine causes neurological and pathological phenotypes mimicking Amyotrophic Lateral Sclerosis (ALS): The first step towards an experimental model for sporadic ALS. Environ. Toxicol. Pharmacol..

[91] De Munck E., Muñoz-Sáez E., Miguel B.G., Solas M.T., Martínez A., Arahuetes R.M. (2015). Morphometric and neurochemical alterations found in l-BMAA treated rats. Environ. Toxicol. Pharmacol..

[92] Esterhuizen-Londt M., Wiegand C., Downing T.G. (2015). β-N-methylamino-l-alanine (BMAA) uptake by the animal model, *Daphnia magna* and subsequent oxidative stress. Toxicon.

[93] Muñoz-Sáez E., de Munck García E., Arahuetes Portero R.M., Vicente F., Ortiz-López F.J., Cantizani J., Miguel B.G. (2015). Neuroprotective role of sphingosine-1-phosphate in L-BMAA treated neuroblastoma cells (SH-SY5Y). Neurosci. Lett..

[94] Muñoz-Sáez E., de Munck García E., Arahuetes Portero R.M., Martínez A., Solas Alados M.T., Miguel B.G. (2015). Analysis of β-N-methylamino-l-alanine (L-BMAA) neurotoxicity in rat cerebellum. NeuroToxicology.

[95] Shen H., Kim K., Oh Y., Yoon K.S., Baik H.H., Kim S.S., Ha J., Kang I., Choe W. (2016). Neurotoxin β-N-methylamino-L-alanine induces endoplasmic reticulum stress-mediated neuronal apoptosis. Mol. Med. Rep..

[96] Cox P.A., Davis D.A., Mash D.C., Metcalf J.S., Banack S.A. (2016). Dietary exposure to an environmental toxin triggers neurofibrillary tangles and amyloid deposits in the brain. Proc. R. Soc. B Biol. Sci..

[97] Brooke-Jones M., Gáliková M., Dircksen H. (2018). Cyanobacterial neurotoxin beta-methyl-amino-l-alanine affects dopaminergic neurons in optic ganglia and brain of *Daphnia magna*. Toxins.

[98] Brenner E.D., Martinez-Barboza N., Clark A.P., Liang Q.S., Stevenson D.W., Coruzzi G.M. (2000). *Arabidopsis* mutants resistant to S(+)-beta-methyl-alpha, beta-diaminopropionic acid, a cycad-derived glutamate receptor agonist. Plant Physiol..

[99] Brenner E.D., Stahlberg R., Mancuso S., Vivanco J., Baluška F., Van Volkenburgh E. (2006). Plant neurobiology: An integrated view of plant signaling. Trends Plant Sci..

[100] Brenner E.D., Feinberg P., Runko S., Coruzzi G.M. (2009). A mutation in the *Proteosomal Regulatory Particle AAA-ATPase-3* in *Arabidopsis* impairs the light-specific hypocotyl elongation response elicited by a glutamate receptor agonist, BMAA. Plant Mol Biol..

[101] Esterhuizen M., Pflugmacher S., Downing T.G. (2011). β-N-Methylamino-l-alanine (BMAA) uptake by the aquatic macrophyte *Ceratophyllum demersum*. Ecotoxicol. Environ. Saf..

[102] Downing S., Esterhuizen-Londt M., Grant Downing T. (2015). β-N-methylamino-L-alanine (BMAA) metabolism in the aquatic macrophyte *Ceratophyllum demersum*. Ecotoxicol. Environ. Saf..

[103] Lage S., Ström L., Godhe A., Rydberg S. (2016). The effect of exogenous β-N-methylamino-l-alanine (BMAA) on the diatoms *Phaeodactylum tricornutum* and *Thalassiosira weissflogii*. Harmful Algae.

[104] Berntzon L., Erasmie S., Celepli N., Eriksson J., Rasmussen U., Bergman B. (2013). BMAA inhibits nitrogen fixation in the cyanobacterium *Nostoc* sp. PCC 7120. Mar. Drugs.

[105] Popova A., Rasmussen U., Semashko T., Govorun V., Koksharova O. (2018). Stress effects of cyanotoxin β-methylamino-L-alanine (BMAA) on cyanobacterial heterocyst formation and functionality *Environ*. Microbiol. Rep..

[106] Popova A., Semashko T., Kostina N., Rasmussen U., Govorun V., Koksharova O. (2018). The cyanotoxin BMAA induces heterocyst specific gene expression in *Anabaena* sp. PCC 7120 under repressive conditions. Toxins.

[107] Koksharova O.A., Butenko I.O., Pobeguts O.V., Safronova N.A., Govorun V.M. (2020). The first proteomic study of *Nostoc* sp. PCC 7120 exposed to cyanotoxin BMAA under nitrogen starvation. Toxins.

[108] Koksharova O.A., Butenko I.O., Pobeguts O.V., Safronova N.A., Govorun V.M. (2020). Proteomic Insights into Starvation of Nitrogen-Replete Cells of *Nostoc* sp. PCC7120 under BMAA Treatment. Toxins.

[109] Koksharova O.A., Butenko I.O., Pobeguts O.V., Safronova N.A., Govorun V.M. (2021). β-N-Methylamino-L-Alanine (BMAA) Causes Severe Stress in *Nostoc* sp. PCC 7120 Cells under Diazotrophic Conditions: A Proteomic Study. Toxins.

[110] Vergou Y., Touraki M., Paraskevopoulou A., Triantis T.M., Hiskia A., Gkelis S. (2020). β-Ν-Methylamino-L-alanine interferes with nitrogen assimilation in the cyanobacterium, non-BMAA producer, *Synechococcus* sp. TAU-MAC 0499. Toxicon.

[111] Wang Z.-Q., Wang S., Zhang J.-Y., Lin G.-M., Gan N., Song L., Zeng X., Zhang C.-C. (2020). The Proposed Neurotoxin β-*N*-Methylamino-L-Alanine (BMAA) Is Taken up through Amino-Acid Transport Systems in the Cyanobacterium *Anabaena* PCC 7120. Toxins.

[112] Banack S.A., Caller T.A., Stommel E.W. (2010). The cyanobacteria derived toxin beta-N-methylamino-L-alanine and amyotrophic lateral sclerosis. Toxins.

[113] Xie X., Basile M., Mash D.C. (2013). Cerebral uptake and protein incorporation of cyanobacterial toxin β-N-methylamino-L-alanine. Neuroreport.

[114] Glover W.B., Mash D.C., Murch S.J. (2014). The natural non-protein amino acid N-β-methylamino-L-alanine (BMAA) is incorporated into protein during synthesis. Amino Acids.

[115] Karlsson O., Bergquist J., Andersson M. (2014). Quality measures of imaging mass spectrometry aids in revealing long-term striatal protein changes induced by neonatal exposure to the cyanobacterial toxin β-N-methylamino-L-alanine (BMAA). Mol. Cell Proteom..

[116] Van Onselen R., Downing T.G. (2018). BMAA-protein interactions: A possible new mechanism of toxicity. Toxicon.

[117] Dunlop R.A., Cox P.A., Banack S.A., Rodgers K.J. (2013). The non-protein amino acid BMAA is misincorporated into human proteins in place of L-serine causing protein misfolding and aggregation. PLoS ONE.

[118] Dunlop R.A., Guillemin G.J. (2019). The Cyanotoxin and non-protein amino acid β-methylamino-L-alanine (L-BMAA) in the food chain: Incorporation into proteins and its impact on human health. Neurotox. Res..

[119] Proctor E.A., Mowrey D.D., Dokholyan N.V. (2019). β-Methylamino-L-alanine substitution of serine in SOD1 suggests a direct role in ALS etiology. PLOS Comput. Biol..

[120] Korn A., Höfling C., Zeitschel U., Krueger M., Roßner S., Huster D. (2020). Incorporation of the Non-Proteinaceous Amino Acid β-Methyl-Amino-Alanine Affects Amyloid β Fibril Properties and Toxicity. ACS Chem. Neurosci..

[121] Han N.C., Bullwinkle T.J., Loeb K.F., Faull K.F., Mohler K., Rinehart J., Ibba M. (2020). The mechanism of β-N-methylamino-L-alanine inhibition of tRNA aminoacylation and its impact on misincorporation. J. Biol. Chem..

[122] Beri J., Nash T., Martin R.M., Bereman M.S. (2017). Exposure to BMAA mirrors molecular processes linked to neurodegenerative disease. Proteomics.

[123] Cui Z., Zhang Y., Inoue H., Yogo S., Hirasawa E. (2013). Purification and molecular analysis of a monoamine oxidase isolated from *Narcissus tazetta*. Biosci. Biotechnol. Biochem..

[124] Silva D.F., Candeias E., Esteves A.R., Magalhães J.D., Ferreira I.L., Nunes-Costa D., Rego A.C., Empadinhas N., Cardoso S.M. (2020). Microbial BMAA elicits mitochondrial dysfunction, innate immunity activation, and Alzheimer’s disease features in cortical neurons. J. Neuroinflamm..

[125] Soto T., Buzzi E.D., Rotstein N.P., German O.L., Politi L.E. (2021). Damaging effects of BMAA on retina neurons and Müller glial cells. Exp. Eye Res..

[126] Nunn P.B., O’Brien P., Pettit L.D., Pyburn S.I. (1989). Complexes of zinc, copper, and nickel with the nonprotein amino acid L-α-amino-β-methylaminopropionic acid: A naturally occurring neurotoxin. J. Inorg. Biochem..

[127] Weiss J.H., Sensi S.L. (2000). Ca^2+^,Zn^2+^ permeable AMPA or kainate receptors: Possible key factors in selective neurodegeneration. Trends Neurosci..

[128] Pochwat B., Nowak G., Szewczyk B. (2015). Relationship between Zinc (Zn2+) and Glutamate Receptors in the Processes Underlying Neurodegeneration. Neural Plast..

[129] Li X., Du X., Ni J. (2019). Zn^2+^ Aggravates Tau Aggregation and Neurotoxicity. Int. J. Mol. Sci..

[130] Diaz-parga P., Goto J.J., Krishnan V.V. (2020). On the differential roles of Mg^2+,^ Zn^2+^, and Cu^2+^ in the equilibrium of β-N-methyl-amino-L-alanine (BMAA) and its carbamates. Neurotox. Res..

[131] Lepoutre A., Milliote N., Bonnard M., Palos-Ladeiro M., Rioult D., Bonnard I., Bastien F., Faassen E., Geffard A., Lance E. (2018). Genotoxic and Cytotoxic Effects on the Immune Cells of the Freshwater Bivalve Dreissena polymorpha Exposed to the Environmental Neurotoxin BMAA. Toxins.

[132] Gerić M., Gajski G., Domijan A.-M., Garaj-Vrhovac V., Filipič M., Žegura B. (2018). Genotoxic effects of neurotoxin ß-N-methylamino-L-alanine in human peripheral blood cells. Chemosphere.

[133] Spencer P.S., Palmer V.S., Kisby G.E. (2018). Cycad β-N-methylamino-L-alanine (BMAA), methylazoxymethanol, genotoxicity, and neurodegeneration. Toxicon..

[134] Pierozan P., Cattani D., Karlsson O. (2020). Hippocampal neural stem cells are more susceptible to the neurotoxin BMAA than primary neurons: Effects on apoptosis, cellular differentiation, neurite outgrowth, and DNA methylation. Cell Death Dis..

[135] Polsky F.I., Nunn P.B., Bell E.A. (1972). Distribution and toxicity of alpha-amino-beta-methylaminopropionic acid. Fed. Proc..

[136] Rodgers K.J. (2014). Non-protein amino acids and neurodegeneration: The enemy within. Exp. Neurol..

[137] Davis D.A., Garamszegi S.P., Banack S.A., Dooley P.D., Coyne T.M., McLean D.W., Rotstein D.S., Mash D.C., Cox P.A. (2021). BMAA, Methylmercury, and Mechanisms of Neurodegeneration in Dolphins: A Natural Model of Toxin Exposure. Toxins.

[138] Duncan M.W., Villacreses N.E., Pearson P.G., Wyatt L., Rapoport S.I., Kopin I.J., Markey S.P., Smith Q.R. (1991). 2-Amino-3-(methylamino)-propanoic acid (BMAA) pharmacokinetics and blood-brain barrier permeability in the rat. J. Pharmacol. Exp. Ther..

[139] Berntzon L., Ronnevi L.O., Bergman B., Eriksson J. (2015). Detection of BMAA in the human central nervous system. Neuroscience.

[140] Frøyset A.K., Khan E.A., Fladmark K.E. (2016). Quantitative proteomics analysis of zebrafish exposed to sub-lethal dosages of β-methyl-amino-L-alanine (BMAA). Sci. Rep.

[141] Goto J.J., Koenig J.H., Ikeda K. (2012). The physiological effect of ingested β-N-methylamino-L-alanine on a glutamatergic synapse in an in vivo preparation. Comp. Biochem. Physiol. C Toxicol. Pharm..

[142] Okle O., Rath L., Galizia C.G., Dietrich D.R. (2013). The cyanobacterial neurotoxin beta-N-methylamino-l-alanine (BMAA) induces neuronal and behavioral changes in honeybees. Toxicol. Appl. Pharm..

[143] Rakonczay Z., Matsuoka Y., Giacobini E. (1991). Effects of L-beta-N-methylamino-Lalanine (L-BMAA) on the cortical cholinergic and glutamatergic systems of the rat. J Neurosci Res.

[144] Allen C.N., Omelchenko I., Ross S.M., Spencer P. (1995). The neurotoxin, β-N-methylamino-l-alanine (BMAA) interacts with the strychnine-insensitive glycine modulatory site of the N-methyl-d-aspartate receptor. Neuropharmacology.

[145] Brownson D.M., Mabry T.J., Leslie S.W. (2002). The cycad neurotoxic amino acid, beta-N-methylamino-L-alanine (BMAA), elevates intracellular calcium levels in dissociated rat brain cells. J. Ethnopharmacol..

[146] Rao S.D., Banack S.A., Cox P.A., Weiss J.H. (2006). BMAA selectively injures motor neurons via AMPA/kainate receptor activation. Exp. Neurol..

[147] Lopicic S., Nedeljkov V., Cemerikic D. (2009). Augmentation and ionic mechanism of effect of beta-N-methylamino-L-alanine in presence of bicarbonate on membrane potential of Retzius nerve cells of the leech *Haemopis sanguisuga*. Comp. Biochem. Physiol. A Mol. Integr. Physiol..

[148] Chiu A., Gehringer M., Braidy N., Guillemin G., Welch J.H., Neilan B.A. (2013). Gliotoxicity of the cyanotoxin, β-methyl-amino-L-alanine (BMAA). Sci. Rep..

[149] Dennison K.L., Spalding E.P. (2000). Glutamate-Gated Calcium Fluxes in Arabidopsis. Plant Physiol..

[150] Davenport R. (2002). Glutamate receptors in plants. Ann. Bot..

[151] Kwaaitaal M., Huisman R., Maintz J., Reinstädler A., Panstruga R. (2011). Ionotropic glutamate receptor (iGluR)-like channels mediate MAMP-induced calcium influx in *Arabidopsis thaliana*. Biochem. J..

[152] Price M.B., Jelesko J., Okumoto S. (2012). Glutamate Receptor Homologs in Plants: Functions and Evolutionary Origins. Front. Plant Sci..

[153] Forde B.G., Roberts M.R. (2014). Glutamate receptor-like channels in plants: A role as amino acid sensors in plant defence?. F1000Prime Rep..

[154] De Bortoli S., Teardo E., Szabò I., Morosinotto T., Alboresi A. (2016). Evolutionary insight into the ionotropic glutamate receptor superfamily of photosynthetic organisms. Biophys. Chem..

[155] Li Y.H., Yu X.Z., Mo L.Y., Lin Y.J., Zhang Q. (2019). Involvement of glutamate receptors in regulating calcium influx in rice seedlings under Cr exposure. Ecotoxicology.

[156] Lazebny O.E., Lazebnaya I.V., Koksharova O.A., A. Troitsk A., Rusin L. Phylogenetic Analysis of Cyanobacterial Glutamate-like Receptors: The First Overlook. Proceedings of the 5th Moscow International Conference “Molecular Phylogenetics and Biodiversity Biobanking”.

[157] Van Onselen R., Cook N.A., Phelan R.R., Downing T.G. (2015). Bacteria do not incorporate β-N-methylamino-l-alanine into their proteins. Toxicon.

[158] Novak M., Hercog K., Žegura B. (2016). Assessment of the mutagenic and genotoxic activity of cyanobacterial toxin beta-N-methyl-amino-L-alanine in Salmonella typhimurium. Toxicon.

[159] Downing S., Van de Venter M., Downing T.G. (2012). The effect of exogenous β-N-methylamino-L-alanine on the growth of *Synechocystis* PCC 6803. Microb. Ecol.

[160] Richter K.E., Mena E.E. (1989). L-beta-methylaminoalanine inhibits [3H]glutamate binding in the presence of bicarbonate ions. Brain Res..

[161] Diaz-parga P., Goto J.J., Krishnan V.V. (2018). Chemistry and chemical equilibrium dynamics of BMAA and its carbamate adducts. Neurotox. Res..

[162] Nunn P.B., Ponnusamy M. (2009). β-N-methylaminoalanine (BMAA): Metabolism and metabolic effects in model systems and in neural and other tissues of the rat in vitro. Toxicon.

[163] Liu X., Rush T., Zapata J., Lobner D. (2009). β-N-methylamino-L-alanine induces oxidative stress and glutamate release through action on system Xc(−). Exp. Neurol.

[164] Herrero A., Stavans J., Flores E. (2016). The multicellular nature of filamentous heterocyst-forming cyanobacteria. FEMS Microbiol. Rev..

[165] Rippka R., Deruelles J., Waterbury J.B., Herdman M., Stanier R.Y. (1979). Generic assignments, strain histories and properties of pure cultures of cyanobacteria. J. Gen. Microbio..

[166] Kaneko T., Nakamura Y., Wolk C.P., Kuritz T., Sasamoto S., Watanabe A., Iriguchi M., Ishikawa A., Kawashima K., Kimura T. (2001). Complete genomic sequence of the filamentous nitrogen-fixing Cyanobacterium *Anabaena* sp. strain PCC 7120. DNA Res..

[167] Castenholz R.W. (2001). Phylum BX. Cyanobacteria (Oxygenic Photosynthetic Bacteria). Bergey’s Manual® of Systematic Bacteriology, Vol. 1: The Archaea and the Deeply Branching and Phototrophic Bacteria.

[168] Singh H.N., Rai U.N., Rao V.V., Bagchi S.N. (1983). Evidence for ammonia as an inhibitor of heterocyst and nitrogenase formation in the cyanobacterium Anabaena cycadeae. Biochem. Biophys. Res. Commun..

[169] Olney J.W., Zorumski C., Price M.T., Labruyere J. (1990). L-cysteine, a bicarbonate-sensitive endogenous excitotoxin. Science.

[170] Chen C.H., Flory W., Koeppe R.E. (1972). Variation of neurotoxicity of L- and D-2,4-diaminobutyric acid with route of administration. Toxicol. Appl. Pharm..

[171] Rawson D.M. (1985). The effects of exogenous amino-acids on growth and nitrogenase activity in the cyanobacterium *Anabaena cylindrica* PCC 7122. J. Gen. Microbiol..

[172] Weiss J.H., Choi D.W. (1988). β-N-methylamino-L-alanine neurotoxicity: Requirement for bicarbonate as a cofactor. Science.

[173] Forchhammer K., Selim K.A. (2019). Carbon/nitrogen homeostasis control in cyanobacteria. FEMS Microbiol. Rev..

[174] Fessenden R.J., Fessenden J.S., Fessenden J.S. (1998). Organic Chemistry.

[175] Gubisne-Haberle D., Hill W., Kazachkov M., Richardson J.S., Yu P.H. (2012). Protein cross-linkage induced by formaldehyde derived from semicarbazide-sensitive amine oxidase-mediated deamination of methylamine. J. Pharmacol. Exp. Ther..

[176] Boomsma F., Van Dijk J., Bhaggoe U.M., Bouhuizen A.M., Van den Meiracker A.H. (2000). Variation in semicarbazide-sensitive amine oxidase activity in plasma and tissues of mammals. Comp. Biochem. Physiol. C Toxicol. Pharmacol..

[177] Percival F.W., Purves W.K. (1974). Multiple amine oxidases in cucumber seedlings. Plant Physiol..

[178] Takser L., Benachour N., Husk B., Cabana H., Gris D. (2016). Cyanotoxins at low doses induce apoptosis and inflammatory effects in murine brain cells: Potential implications for neurodegenerative diseases. Toxicol. Rep..

[179] Santucci S., Zsürger N., Chabry J. (2009). β- N -methylamino-l-alanine induced in vivo retinal cell death. J. Neurochem..

[180] Azam F., Worden A.Z. (2004). Microbes, Molecules, and Marine Ecosystems. Science.

[181] Pohnert G., Amsler C.D. (2008). Influence of Algal Secondary Metabolites on Plankton Community Structure. Algal Chemical Ecology.

[182] Koksharova O.A., Safronov N.A. (2022). The effects of bacterial secondary metabolites on photosynthesis in microalgae cells. Biophys. Rev..

[183] Warshan D., Espinoza J.L., Stuart R.K., Richter R.A., Kim S.-Y., Shapiro N., Woyke T., Kyrpides N., Barry K., Singan V. (2017). Feathermoss and epiphytic Nostoc cooperate differently: Expanding the spectrum of plant-cyanobacteria symbiosis. ISME J..

[184] Kim S.Y., Rasmussen U., Rydberg S. (2022). Effect and function of β-N-methylamino-L-alanine in the diatom *Phaeodactylum tricornutum*. Sci. Total Environ..

[185] Nieves-Morión M., Flores E., Foster R.A. (2020). Predicting substrate exchange in marine diatom-heterocystous cyanobacteria symbioses. Environ. Microbiol..

[186] Grady E.N., MacDonald J., Liu L., Richman A., Yuan Z.-C. (2016). Current knowledge and perspectives of Paenibacillus: A review. Microb. Cell Factories.

[187] Ageitos J.M., Sánchez-Pérez A., Calo-Mata P., Villa T.G. (2017). Antimicrobial peptides (AMPs): Ancient compounds that represent novel weapons in the fight against bacteria. Biochem. Pharmacol..

[188] Martin R.M., Bereman M.S., Marsden K.C. (2020). Exposure to a mixture of BMAA and MCLR synergistically modulates behavior in larval zebrafish while exacerbating molecular changes related to neurodegeneration. bioRxiv.

[189] Kazemi Shariat Panahi H., Dehhaghi M., Heng B., Lane D.J.R., Bush A.I., Guillemin G.J., Tan V.X. (2022). Neuropathological Mechanisms of *β*-N-Methylamino-L-Alanine (BMAA) with a Focus on Iron Overload and Ferroptosis. Neurotox. Res..

